# DN203316, a novel PPARδ agonist, suppresses ferroptotic signaling and fibrogenesis in metabolic dysfunction-associated steatohepatitis

**DOI:** 10.1038/s12276-026-01740-0

**Published:** 2026-06-05

**Authors:** Ye Jin Kim, Jina Kim, Da Young An, Mihyang Park, Gui-Hwa Jeong, Jonghwa Jin, Mi Kyung Kim, Jungwook Chin, Yeon-Kyung Choi, Keun-Gyu Park

**Affiliations:** 1 Department of Internal Medicine, School of Medicine, Kyungpook National University, Kyungpook National University Hospital, Daegu, Republic of Korea; 2 Research Institute of Aging and Metabolism, Kyungpook National University, Daegu, Republic of Korea; 3 New Drug Development Center, Daegu-Gyeongbuk Medical Innovation Foundation, Daegu, Republic of Korea; 4 Department of Biomedical Science, Kyungpook National University, Daegu, Republic of Korea; 5 Departmentof Internal Medicine, CHA Gumi Medical Center, CHA University, Gumi, Republic of Korea; 6 Department of Internal Medicine, Keimyung University School of Medicine, Daegu, South Korea; 7 Cureverse, KIST, Seoul, Republic of Korea; 8 Department of Internal Medicine, School of Medicine, Kyungpook National University, Kyungpook National University Chilgok Hospital, Daegu, Republic of Korea

**Keywords:** Mechanisms of disease, Drug development

## Abstract

Recently, ferroptosis has emerged as a pathogenic mechanism that drives metabolic dysfunction-associated steatohepatitis (MASH); however, the upstream triggers and their relevance to fibrosis remain poorly understood. Here we identified dietary cholesterol-induced ferroptosis and the downregulation of peroxisome proliferator-activated receptor delta (PPARδ) as central drivers of MASH pathogenesis. To investigate this, human liver samples and cholesterol-enriched dietary murine models of MASH were examined in parallel with mechanistic studies in hepatocytes and hepatic stellate cells (HSCs). Cholesterol-induced MASH was associated with pronounced hepatic lipid peroxidation and the selective downregulation of PPARδ. The loss of PPARδ disrupted redox homeostasis and sensitized hepatocytes to ferroptosis, whereas exosomal double-stranded DNA released from ferroptotic hepatocytes activated STING–TBK1–IRF3 and induced expression of profibrotic genes in HSCs. These effects were reversed by either overexpression of hepatocyte-specific PPARδ or pharmacologic treatment with DN203316, a novel and highly selective PPARδ agonist. In vivo, DN203316 mitigated ferroptosis, inflammation and fibrosis without inducing metabolic derangement. These findings were substantiated by clinical data demonstrating a marked increase in lipid peroxidation and STING-driven HSC activation in liver tissues from patients with MASH. In conclusion, PPARδ is a key regulator of cholesterol-induced ferroptosis and exosome-mediated fibrogenic signaling in MASH. DN203316 offers a promising therapeutic strategy to suppress ferroptosis, disrupt hepatocyte–HSC crosstalk and attenuate disease progression.

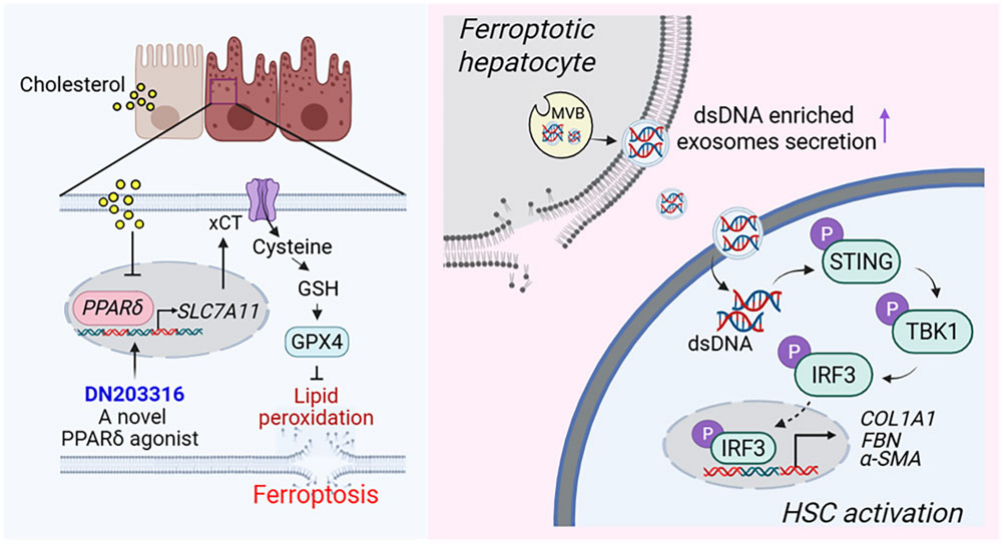

## Cholesterol triggers ferroptosis and fibrosis in liver disease

Metabolic dysfunction-associated steatohepatitis (MASH) is a severe form of liver disease characterized by inflammation and fibrosis, driven by complex metabolic and cellular interactions. This study identifies ferroptosis, a form of cell death linked to lipid peroxidation, as a key mechanism in MASH progression, highlighting the role of peroxisome proliferator-activated receptor delta (PPARδ) in regulating this process. Researchers used human liver samples and mouse models to demonstrate that cholesterol-induced ferroptosis in hepatocytes leads to the release of exosomal DNA, activating fibrogenic pathways in hepatic stellate cells. They found that the downregulation of PPARδ exacerbates ferroptosis, whereas its activation, through genetic overexpression or the novel agonist DN203316, mitigates liver damage by enhancing antioxidant defenses. These findings suggest that targeting PPARδ could offer a new therapeutic strategy for MASH, with DN203316 showing potential as a treatment to reduce liver fibrosis and inflammation. Future research could explore the broader implications of PPARδ modulation in metabolic liver diseases. This summary was initially drafted using artificial intelligence, then revised and fact-checked by the author.

## Introduction

Metabolic dysfunction-associated steatohepatitis (MASH) is a pivotal and increasingly prevalent form of metabolic dysfunction-associated steatotic liver disease, now recognized as the leading cause of chronic liver disease and liver-related mortality worldwide^1–5^. Unlike simple steatosis, MASH is distinguished by a constellation of pathological features, including hepatocyte ballooning, lobular inflammation and progressive fibrosis, which together drive the transition toward advanced liver dysfunction and cirrhosis^6–8^. The pathogenesis of MASH is multifaceted, involving metabolic derangements such as insulin resistance and lipid accumulation and the interplay between oxidative stress, mitochondrial dysfunction and immune-mediated injury^9–13^. Accumulating evidence indicates that hepatocyte cell death, via both apoptotic and nonapoptotic pathways, serves as a central trigger for inflammatory and fibrogenic cascades that drive disease progression^14–18^. This complexity underscores the urgent need to elucidate the molecular mechanisms governing cell fate and tissue remodeling in MASH, as current therapeutic options remain limited.

Ferroptosis, an iron-dependent and nonapoptotic form of regulated cell death, has emerged as a critical contributor to MASH pathogenesis^19,20^. Mechanistically, ferroptosis is distinct from apoptosis, necrosis and pyroptosis because it is driven by excessive lipid peroxidation and the failure of antioxidant defenses, particularly glutathione peroxidase 4 (GPX4)^21–24^. The central role of the liver in iron metabolism, as well as its susceptibility to oxidative stress, make it particularly vulnerable to ferroptotic injury^24,25^. In the context of MASH, hepatocyte ferroptosis contributes not only to parenchymal injury but also to establishment of a proinflammatory and immunosuppressive milieu that facilitates activation and fibrosis of hepatic stellate cells (HSC)^26,27^. Although various modes of hepatocyte death, including apoptosis and nonapoptotic mechanisms, are implicated in initiating inflammatory and fibrogenic responses, ferroptosis represents a distinct and potent driver of these pathogenic cascades. So far, there are no approved pharmacologic therapies that specifically target ferroptosis in MASH, underscoring the need to elucidate upstream regulatory pathways that could inform the development of novel interventions.

Peroxisome proliferator-activated receptor delta (PPARδ) is a nuclear receptor that orchestrates expression of genes involved in lipid metabolism, mitochondrial function and inflammation, processes that are fundamental to metabolic homeostasis^28–31^. Although PPARα and PPARγ have established roles in regulating glutathione (GSH) metabolism and ferroptosis via the modulation of GSH and GPX4^32,33^, the specific involvement of PPARδ in ferroptotic processes remains largely unexplored. The downregulation of PPARδ is observed in several metabolic disorders, with emerging evidence pointing to its deficiency as a potential amplifier of oxidative damage; however, the direct relationship between PPARδ signaling, ferroptotic cell death in hepatocytes and the consequent impact on liver fibrosis in MASH has yet to be fully delineated. Thus, deciphering the interplay between PPARδ and ferroptosis may unlock new avenues for therapeutic intervention in metabolic and degenerative liver diseases.

Here, we investigated whether the downregulation of PPARδ in hepatocytes promotes ferroptosis and contributes to the activation of HSCs and liver fibrosis in MASH. We also examined the therapeutic potential of DN203316, a novel selective PPARδ agonist, as a suppressor of hepatocyte ferroptosis and liver fibrosis in diet-induced MASH models. Our findings identify a previously unrecognized PPARδ–ferroptosis axis that drives pathogenesis of MASH and offer a new pharmacologic strategy for therapeutic intervention.

## Methods

### Patients and specimens

Samples of human liver tissue were obtained from patients with MASH (*n* = 5) and without MASH (*n* = 5) who underwent surgical procedures, including bariatric or oncologic surgery at Keimyung University Dongsan Hospital in Daegu, Korea. In addition, an independent cohort of patients with MASH (*n* = 5) and without MASH (*n* = 5) was included for analysis. The study was approved by the Institutional Review Board of Keimyung University Dongsan Hospital (Institutional Review Board no. 2024-06-033 and DSMC 2015-01-015), and written informed consent was obtained from all participants.

### Animal models

Male C57BL/6 mice were obtained from Jackson Laboratory (Dooyeol Biotech). To establish a short-term hepatic fibrosis model, male C57BL/6J mice (8–10 weeks old) were fed a cholesterol-enriched diet (CED) containing 2.5% (w/w) cholesterol for 6 days. Control mice were fed a standard chow diet under the same conditions. To induce MASH, mice (5–6 weeks old) were fed a high-fat, high-cholesterol (HFHC) diet or a normal diet for 14 weeks. To achieve liver-specific overexpression, the full-length murine PPARδ complementary DNA (cDNA) with an mCherry tag was cloned into an adeno-associated virus 8 (AAV8) vector under the control of the thyroxine-binding globulin (TBG) promoter (VectorBuilder). The overexpression of PPARδ was achieved by injection of AAV8-TBG-PPARδ (1 × 10^11^ vg/ml) into C57BL/6J mice through the tail vein. AAV8 mCherry was used as a control. Mice were fed a HCHF diet for 9 weeks before the first injection. Mice received weekly tail vein injections of AAV8-TGB-PPARδ for five consecutive weeks, starting at week 9. At the experimental endpoint, the mice were killed, and tissue samples were collected, sectioned and snap-frozen immediately in liquid nitrogen for further analysis. To evaluate the therapeutic efficacy of DN203316, a novel selective PPARδ agonist, HCHF-induced MASH model mice received an intraperitoneal injection at a dose of 3 mg/kg body weight (once daily from week 9 to week 14). All animal procedures were performed in accordance with guidelines approved by the Institutional Animal Care and Use Committee of Kyungpook National University (approval no. IACUC-2023-0547).

### Cell culture

The murine hepatocyte cell line AML12 (ATCC) was cultured in DMEM–F12 medium (Gibco) containing 10% fetal bovine serum (FBS), 5 mg/ml insulin, 5 μg/ml transferrin, 5 ng/ml selenium (ITS mix, Thermo Fisher Scienfitic), 40 ng/ml dexamethasone (Sigma-Aldrich) and 1% penicillin–streptomycin (Gibco). The HepG2 (ATCC) cells were cultured in Eagle’s minimum essential medium (ATCC) supplemented with 10% FBS and 1% penicillin–streptomycin. All cells were incubated at 37 °C under an atmosphere containing 5% CO_2_.

### Chemical treatments

DN203316, a novel selective PPARδ agonist, was synthesized as described^34^. Cholesterol, ferrostatin-1 (Fer-1; ferroptosis inhibitor), Z-VAD-FMK (apoptosis inhibitor) and IM-54 (necrosis inhibitor) were purchased from Sigma. The STING inhibitor SN-011 was purchased from MedChem express. Cells were treated with DN203316 (1 μM) or Fer-1 (2 μM), IM-54 (10 μM) or Z-VAD-FMK (20 μM) for 1 h before stimulation with water-soluble cholesterol (100 μg), followed by incubation for 24 h.

### BODIPY staining

For the lipid peroxidation assay, cells were stained for 30 min with 2 μM BODIPY 581/591 C11 (Thermo Fisher Scientific) and washed three times with PBS, and the nuclei were stained with NucBlue Live cell ReadyProbes Reagent (Thermo Fisher Scientific). Lipid peroxidation in human and mouse liver tissues was assessed using the method described by Wang et al.^35^. In brief, after dissection, livers were snap-frozen in liquid nitrogen and stored at −80 °C until use. Frozen liver tissues were transferred to −20 °C at least 30 min before sectioning to equilibrate their temperature with that of the cryostat chamber. Cryosectioned liver tissues (8–10-μm thickness) were air-dried on glass slides for 10 min and fixed for 10 min at room temperature in 4% paraformaldehyde. Sections were washed three times with PBS and incubated (30 min at 37 °C in the dark) with 2 μM BODIPY 494/503 (Thermo Fisher Scientific). After incubation, sections were rinsed with PBS and mounted in antifade mounting medium containing DAPI (H-1200-10, VECTASHIELD with DAPI; Vector Laboratories). Fluorescence intensity was quantified using ImageJ software.

### Histological analysis

Liver tissue samples were removed from mice postmortem, fixed with 4% paraformaldehyde (Biosesang) and embedded in paraffin. The hematoxylin and eosin (H&E) staining of paraffin-embedded sections was carried out using standard procedures. To assess the collagen accumulation, liver tissue sections were stained for 1 h with 0.1% Sirius Red solution in saturated picric acid. After staining, the slides were washed in acidified water (0.5% acetic acid), dehydrated through a graded series of ethanol solutions, cleared in xylene and mounted under coverslips. For immunohistochemistry (IHC) staining, paraffinized liver tissue sections were deparaffinized with xylene and ethanol, and endogenous peroxidase was blocked by treatment for 15 min with 3% H_2_O_2_. Tissues were incubated with primary antibodies specific for PPARδ (Cell Signaling Technology), 4-hydroxynonenal (4-HNE; R&D), α-SMA (Sigma-Aldrich) and Col1a1 (Cell Signaling Technology), all diluted at 1:200. Bound primary antibodies were detected by a horseradish peroxidase (HRP)-conjugated secondary antibody, followed by staining with diaminobenzidine (Liquid DAB + Substrate Chromogen System, Dako). α-SMA and p-STING-positive immunostaining was evaluated using ImageJ software (National Institutes of Health).

### NAS

Formalin-fixed, paraffin-embedded liver biopsies from patients with suspected MASH were histologically evaluated. Disease activity was graded using the nonalcholic fatty liver disease (NAFLD) activity score (NAS; range, 0–8) on the basis of Nonalcoholic Steatohepatitis Clinical Research Network (NASH CRN) criteria, calculated as the sum of scores for steatosis (0–3), lobular inflammation (0–3) and hepatocellular ballooning (0–2), whereas fibrosis was evaluated by Sirius red staining, which highlights collagen deposition.

### Western blot analysis

Proteins were extracted using radioimmunoprecipitation buffer (Thermo Fisher Scientific) supplemented with protease and phosphatase inhibitors, and lysates were clarified by centrifugation at 17,000*g* for 20 min at 4 °C. Protein concentrations were determined using the BCA assay (Thermo Fisher Scientific). Equal amounts of protein were separated by 10% SDS–polyacrylamide gel electrophoresis and transferred to polyvinylidene fluoride membranes (Millipore). The membranes were blocked with 5% skim milk in Tris-buffered saline containing 0.1% Tween 20 and then incubated overnight at 4 °C with appropriate primary antibodies specific for the following proteins: PPARδ, PPARα, PPARγ, xCT, GPX4, COL1A1, p-STING, STING, p-TBK1, TBK1, p-IRF3, IRF3 (all from Cell Signaling Technology), fibronectin (BD Biosciences), α-SMA (Thermo Fisher Scientific), ALOX15, STING, TBK1, IRF3 or p-STING, p-TBK1 and p-IRF3 (Cell Signaling Technology), ACSL4 (Santa Cruz Biotechnology), LPCAT3 (MyBioSource) and SLC3A2 (ABclonal), β-actin (Sigma-Aldrich) and GAPDH (Cell Signaling Technology). After three washes in Tris-buffered saline containing 0.1% Tween 20, the membranes were incubated with HRP-conjugated secondary antibodies (GeneTex). The quantification of band intensity was performed using ImageJ software (National Institutes of Health).

### RNA isolation and rtPCR

Total RNA was extracted from liver tissue or cells using Trizol reagent (Qiagen), and cDNA was synthesized using a RevertAid first strand cDNA synthesis kit (Thermo Fisher Scientific). Real-time PCR (rtPCR) was performed using SYBR green (Qiagen)-based QPCR on a Biorad CFX96 machine. The relative expression of mRNA was calculated from the comparative cycle threshold values relative to GAPDH mRNA. Data are presented as arbitrary units and were calculated by the 2^ΔΔCT^ method. Primer sequences were provided by Integrated DNA Technologies and are listed in Supplementary Table 1.

### Transfection of siRNA

Cells were transfected with control small interfering RNA (siRNA), mouse or human siPPARδ or human siRab27a (Bioneer) using Lipofectamine RNAi MAX (Thermo Fisher Scientific). In brief, cells were seeded overnight (~12 h) in 60-mm dishes at a density of 2 × 10^5^ per well and then incubated for 48 h in 0.2 ml of culture medium containing 6 μl Lipofectamine RNAi MAX and 60 pmol of siRNA.

### GSH assay

Hepatocytes and hepatic GSH levels were measured using GSH-Glo Assays (Promega). GSH levels were normalized to cell or tissue weight.

### Cell viability

The cell viability measured using the CCK-8 kit (Dojindo Laboratories). AML12 and HepG2 cells were seeded in 96-well plates and cultured overnight. Cells were then treated with 100 μg cholesterol, 2 μM Fer-1 (Sigma-Aldrich), DN203316, an apoptosis inhibitor (Z-VAD-FMK; 50 μM) or a necrosis inhibitor (IM5; 50 μM) for the indicated times. At the indicated times, 10 μl CCK-8 was added to each well to measure viability. After 1–2 h, cell viability was determined by measuring optical density at 450 nm.

### PI staining

To evaluate cell death, cells were stained with propidium iodide (PI) (Sigma-Aldrich). In brief, 1 × 10^5^ hepatocytes were plated overnight in 60-mm dishes and then treated for 24 h with cholesterol or DN203316. Next, PI was added to each well for 1 min (room temperature and shielded from light). Cell death was determined by measuring PI fluorescence in the FL3 channel of a BD Accuri C6 flow cytometer (BD Biosciences).

### Isolation of mouse hepatocytes, KCs and stellate cells

Primary hepatocytes, Kupffer cells (KCs) and HSCs were isolated from the indicated mice via portal vein perfusion following anesthesia, as described previously^36^. In brief, the liver was perfused in situ with Ca^2+^-free Hank’s Balanced Salt Solution (Welgene) for 15 min, followed by 100 ml of 0.2% pronase solution and 0.2% collagenase type IV (Worthington) until the liver looked digested and became pale in color. The acquired cell suspension was filtered through a 100-μm pore size mesh nylon filter (Falcon) and then centrifuged at 50*g*. The pellet was collected for use as primary hepatocytes, and the supernatant was collected for further isolation of primary KCs and HSCs by density gradient centrifugation. KCs (F4/80^high^ CD11b^int^) were identified by surface staining, and their purity was confirmed by flow cytometry using a BD Accuri C6 Plus (BD Biosciences). The primary hepatocytes, KCs and HSCs were then cultured in RPMI medium 1640 supplemented with 10% FBS.

### Overexpression of PPARδ using a lentivirus system

Lenti-PPARδ (pLV-mCherry-EF1A-Flag/PPARδ; VectorBuilder) was used to generate stable hepatocyte cell lines overexpressing PPARδ. In brief, HEK293 cells were transfected with the Lenti-PPARδ plasmid using Lipofectamine 3000 (Thermo Fisher Scientific). After 48–72 h, the culture medium containing lentiviral particles was collected and filtered through a 0.45-μm syringe filter. To induce the expression of PPARδ in HepG2 cells, AML12 cells and primary mouse hepatocytes, the cells were infected for 24 h with the collected lenti-PPARδ-containing medium under serum-free conditions.

### Luciferase reporter assays

To assess the effect of cholesterol on PPARδ transcription, AML12 murine hepatocytes were seeded in 24-well plates and transfected with a pEZX-PG04-PPARδ promoter vector (GeneCopoeia). Following transfection, cells were challenged with various cholesterol concentrations for 24 h. In addition, to evaluate whether PPARδ regulates xCT promoter activity, luciferase reporter assays were performed using the pEZX-PG04-xCT construct (GeneCopoeia), containing either the full-length or site-mutated xCT promoter. AML12 cells were cotransfected with the reporter plasmids and a PPARδ expression vector. For both assays, transcriptional activity was quantified by measuring secreted Gaussia luciferase signals in the culture supernatant using the Secrete-Pair Dual Luminescence Assay Kit (GeneCopoeia) according to the manufacturer’s instructions. All bioluminescence data were normalized to alkaline phosphatase (AP) activity to consider variations in transfection efficiency.

### Actinomycin D chase assay

Hepatocytes were treated with cholesterol for 24 h, followed by actinomycin D (5 μg/ml) to inhibit de novo transcription. Cells were harvested at 0, 2, 8 and 24 h post-treatment, and total RNA was isolated using TRIzol reagent. PPARδ mRNA levels were quantified using quantitative rtPCR, normalized to GAPDH.

### Preparation of hepatocyte-derived CM

To generate conditioned medium (CM) from primary hepatocytes, AML12 and HepG2 cells, 3 × 10^6^ cells were seeded into a 150-mm culture dishes. The following day, the medium was replaced with fresh medium, and the hepatocytes were treated with cholesterol for 24 h in the presence or absence of Fer-1, DN203316 or overexpressed PPARδ. The CM was collected and centrifuged at 1,500*g* for 15 min before use.

### Co-culture experiments

Primary HSCs were isolated from normal mice and seeded overnight on Transwell inserts (50,000 per insert; Corning). After adherence, the insert was placed above primary hepatocytes (100,000 cells per well) isolated from normal mice and co-cultured for 24 h. Hepatocyte cells were stimulated (or not) with cholesterol and then co-cultured for 24 h with mouse or human HSCs (mHSC and LX2) in a 0.4-μm Transwell plate (Corning). Primary HSC and LX2 cell lysates were harvested for further experiments.

### Isolation of exosomes from hepatocyte-derived supernatants

Exosomes were isolated from cell culture supernatants by differential ultracentrifugation. In brief, conditioned media were centrifuged for 10 min at 2,000*g*, followed by a further centrifugation for 30 min at 10,000*g*. The supernatant was then passed through a 0.22-μm filter. A final centrifugation at 100,000*g* for 70 min at 4 °C was performed to isolate exosomes, and the collected pellets were washed in PBS, followed by a second round of ultracentrifugation at 100,000*g* for 70 min to purify the exosomes. The pellets were resuspended in stabilization solution and applied to HSCs.

### Measurement of extracellular dsDNA levels

Exosomal double-stranded DNA (dsDNA) levels in exosomes derived from hepatocyte-conditioned media were quantified using the Quant-iT PicoGreen dsDNA Assay Kit (Thermo Fisher Scientific). In brief, samples of conditioned media were mixed with the PicoGreen reagent in a black 96-well plate. Fluorescence intensity was measured using a microplate reader at an excitation wavelength of 480 nm and an emission wavelength of 520 nm. The concentration of dsDNA was determined from a standard curve generated using lambda DNA standards.

### Measurement of mtDNA in exosomes

Exosomes were isolated, and their total DNA was extracted using the AccuPrep Genomic DNA Extraction Kit (Bioneer). The total DNA was subsequently purified via a spin-column method, and its concentration and purity were assessed using a Nanodrop spectrophotometer. Exosomal mitochondrial DNA (mtDNA) was quantified by quantitative PCR targeting a region of the NADH dehydrogenase 1 (*ND1*) gene, with the corresponding primer sequences provided in Supplementary Table 1.

### Digestion of external DNA of exosomes

To determine whether dsDNA (including mtDNA) transferred and taken up by LX2 cells via exosomes activates the STING–TBK1–IRF3 pathway, the exosomal dsDNA was degraded before analysis. In brief, purified exosomes were permeabilized by incubating with 0.1% Triton X-100 (v/v) at room temperature for 10 min with gently mixing. Subsequently, RNase-free DNase I (2 unit/ml) was added, and the mixture was incubated at 37 °C for 30 min to digest any exposed DNA. The treated exosomes were washed with PBS and subsequently used for downstream analyses.

### Detection of anti-AAV8 IgG by ELISA

To evaluate the humoral immune response against the viral vector, serum levels of AAV8-specific IgG were quantified using an Anti-AAV8 antibody enzyme-linked immunosorbent assay (ELISA) kit (Acro Biosystems) according to the manufacturer’s instructions.

### Cytokine measurement

Mouse serum and cell culture supernatants were collected, clarified by centrifugation (3,000*g*, 10 min, 4 °C) and stored at −80 °C until analysis. TNF-α, IL-1β and IL-6 concentrations were quantified using commercially available ELISA kits (BioLegend) according to the manufacturer’s instructions.

### CXCL12 measurement

CXCL12 concentrations were determined using a commercially available ELISA kit (Abclonal) following the manufacturer’s protocol.

### Biochemical analyses of murine liver and serum samples

Liver function was evaluated by measuring serum ALT and AST activity using commercial assay kits from Sigma-Aldrich.

### Statistical analysis

All data are presented as the mean ± s.e.m. and are representative of at least three independent experiments. Statistical analyses were performed using GraphPad 8 software (GraphPad Software). Two-group comparisons were analyzed using unpaired two-tailed Student’s *t*-test, and multiple-group comparisons using one-way analysis of variance (ANOVA) with Dunnett’s or Tukey’s post hoc test as appropriate. *P* < 0.05 was considered statistically significance.

## Results

### Cholesterol-induced ferroptosis and downregulation of PPARδ are key features of MASH

**Fig. 1:**
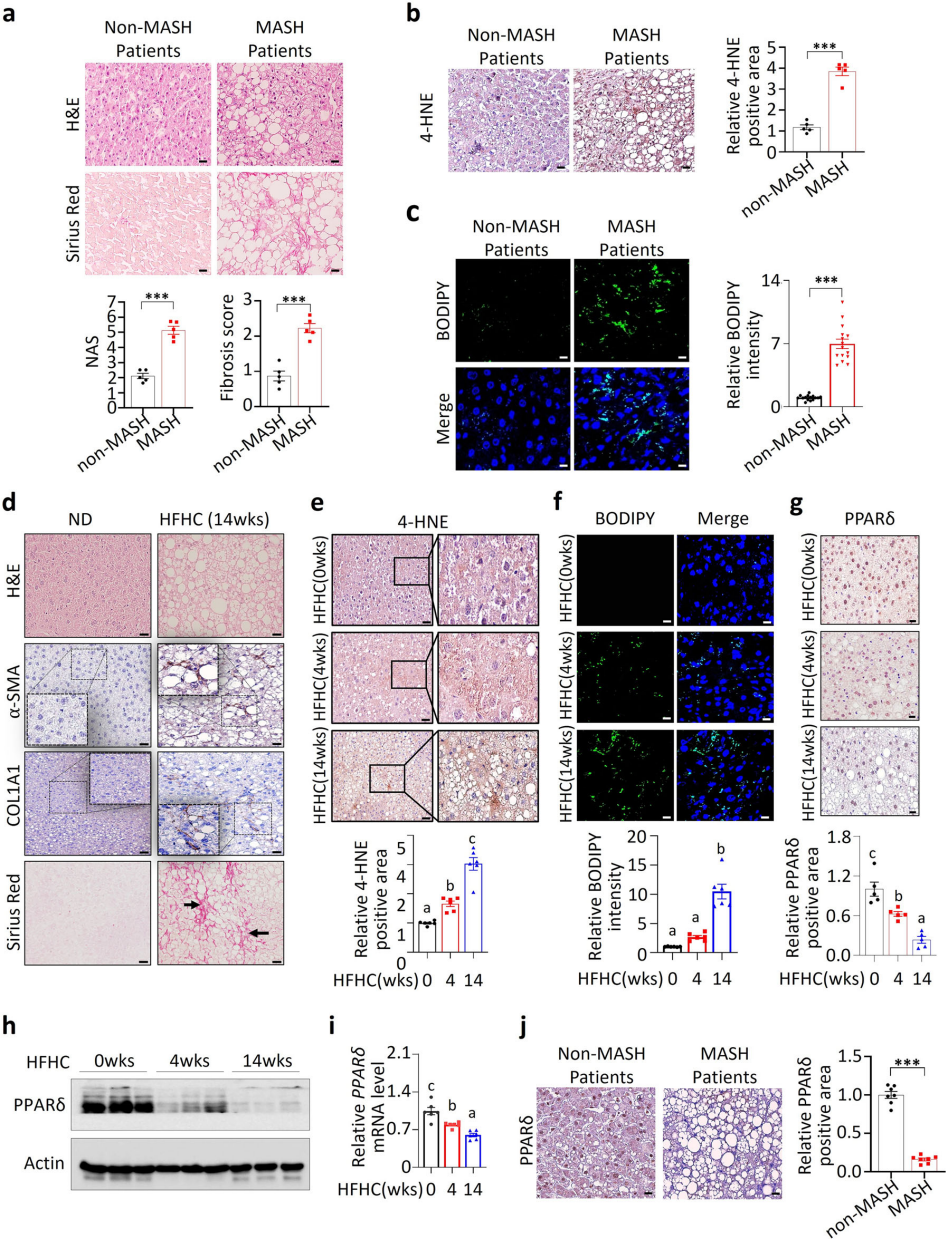
A high-cholesterol diet induces hepatic ferroptosis and suppresses expression of PPARδ. **a** The representative histological images of liver sections from non-MASH patients and patients with MASH stained with H&E and Sirius Red. **b** The IHC for 4-HNE in liver tissues of non-MASH patients and patients with MASH. Right: a quantitative analysis of positive cells. **c** The representative immunofluorescence images of liver sections from patients with MASH, showing lipid peroxidation detected by BODIPY staining. Right: the quantification of BODIPY-positive cells. **d** The histological evaluation (H&E staining and IHC) analysis of fibrotic markers (α-SMA and Col1a1) and Sirius red staining of liver tissues from HFHC diet-induced MASH mice. **e**,**f** The assessment of hepatic lipid peroxidation in HFHC-fed mice: IHC analysis to detect 4-HNE (**e**) and BODIPY staining (**f**). Bottom: a quantitative analysis of positive cells. **g** The IHC analysis of PPARδ expression in liver sections from HFHC-fed mice. Bottom: a quantification of PPARδ-positive cells. **h**, **i** A western blot and quantitative PCR analyses of hepatic PPARδ protein and mRNA levels in HFHC-induced MASH mice. **j** The IHC analysis of PPARδ expression in liver sections from non-MASH patients and patients with MASH. Right: quantitative analysis of positive cells. Data are expressed as the mean ± s.e.m. of three independent experiments. Scale bar, 20 µm. Samples denoted by different letters (**a**,**b** and **c**) vary significantly (*P* < 0.05, one-way ANOVA followed by Tukey’s post hoc test). **P < 0.01, ***P < 0.001.

To determine the clinical relevance of ferroptosis in MASH, we initially evaluated lipid peroxidation in human liver samples from patients with and without MASH. Based on the histologic analysis by H&E and Sirius red staining in liver tissues, the NAS and fibrosis score were significantly elevated in patients with MASH (Fig. 1a and Supplementary Fig. 1a), accompanied by a marked increased 4-HNE immunostaining (Fig. 1b). Using C11-BODIPY fluorescence following a published protocol^37^, we identified a substantial increase in lipid peroxidation in MASH liver tissues, implicating ferroptosis as a histological feature (Fig. 1c). Given that dietary cholesterol is a key driver of MASH pathogenesis^38,39^, we investigated its contribution using two cholesterol-based dietary mouse models. Mice fed a HFHC diet for 14 weeks recapitulated the key features of human MASH, that is, hepatic steatosis, inflammation and fibrosis (Fig. 1d). Next, we used 4-HNE immunostaining and C11-BODIPY fluorescence to further evaluated the role ferroptosis in MASH. Notably, we identified a marked increase in lipid peroxidation in MASH liver tissues, implicating ferroptosis as a histological feature of MASH (Fig. 1e,f). To specifically determine whether the increase in ferroptosis observed with the HFHC diet was attributable to cholesterol itself, we subjected mice to a CED for 6 days. Short-term cholesterol feeding was sufficient to induce significant hepatic ferroptosis, as evidenced by increased C11-BODIPY fluorescence and 4-HNE accumulation compared with chow-fed controls (Supplementary Fig. 1b,c). In parallel, we observed a robust, time-dependent downregulation of PPARδ at both the transcript and protein levels in mice fed either a HFHC or CED diet (Fig. 1g–i and Supplementary Fig. 1d,f). The downregulation of PPARδ was further validated in liver tissues from patients with MASH, as shown by significantly reduced PPARδ immunostaining compared with non-MASH controls (Fig. 1j). Collectively, these findings establish ferroptosis as a hallmark of cholesterol-induced liver injury in MASH, mechanistically linking PPARδ suppression by dietary cholesterol to enhanced ferroptosis susceptibility and disease progression.

### Suppression of PPARδ mediates cholesterol-induced ferroptosis via impaired antioxidant defense

**Fig. 2:**
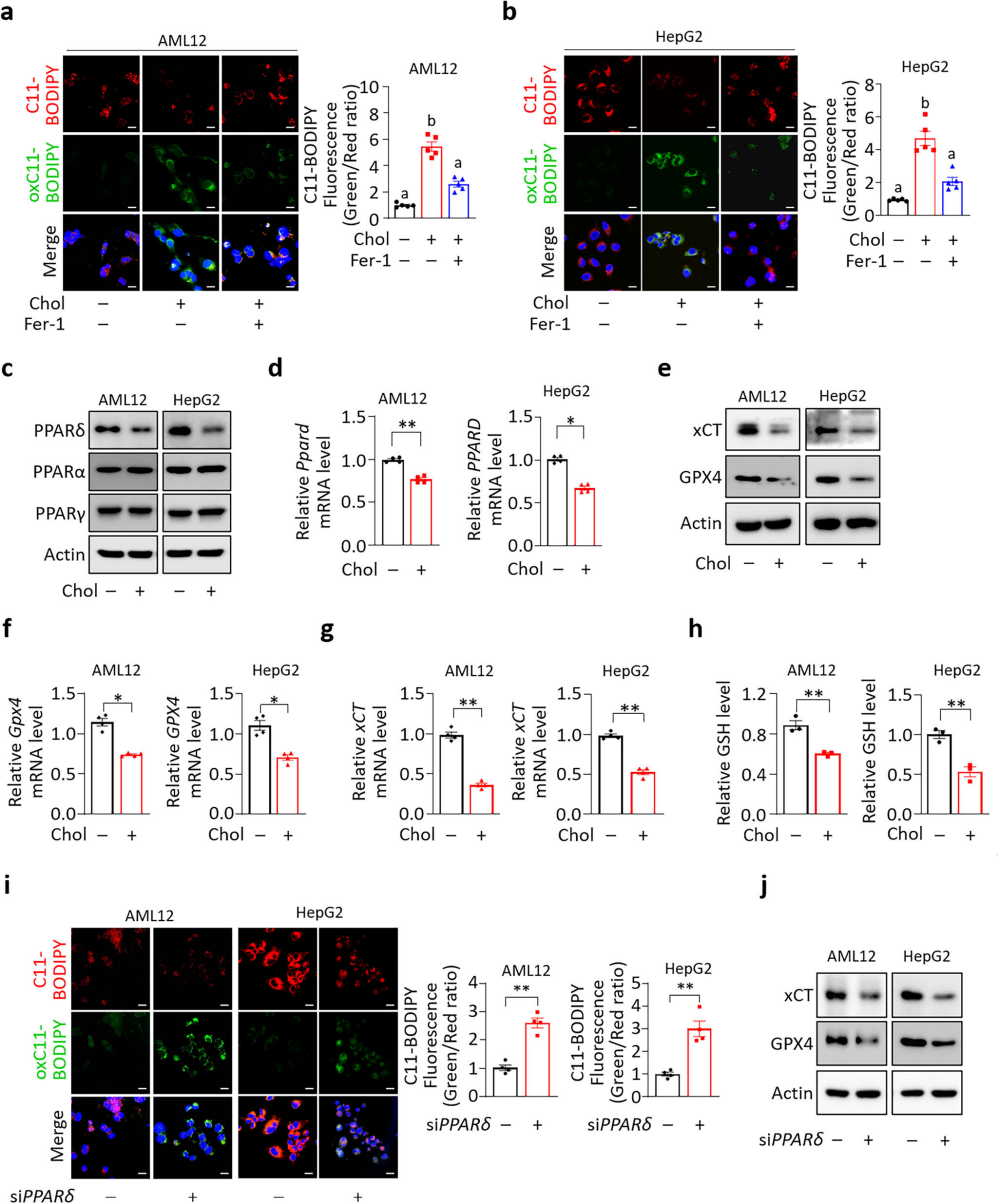
Cholesterol promotes ferroptosis in hepatocytes by suppressing the xCT/GPX4 antioxidant pathway. **a**, **b** Representative immunofluorescence images of lipid peroxidation, as detected by BODIPY staining, in cholesterol-treated AML12 (**a**) and HepG2 (**b**) cells. Right: a quantification of oxC11-BODIPY-positive cells is shown. **c**,**d** The expression of PPAR isoforms (α, γ and δ) at the protein (**c**) and mRNA levels (**d**) in hepatocytes undergoing cholesterol-induced ferroptosis. **e**–**g** The expression of xCT and GPX4 in AML12 and HepG2 cells, as assessed by immunoblotting (**e**) and quantification of mRNA (**f**,**g**). **h** Intracellular GSH levels in AML12 and HepG2 cells cultured with or without cholesterol. **i** Left: the representative BODIPY-stained images of AML12 and HepG2 cells under PPARδ-knockdown conditions. Right: the quantification of oxC11-BODIPY–positive cells. **j** The expression of xCT and GPX4 protein in PPARδ-silenced AML12 and HepG2 cells. The data are expressed as the mean ± s.e.m. of three independent experiments. Scale bar, 20 µm. Samples denoted by different letters (**a** and **b**) vary significantly (*P* < 0.05, one-way ANOVA followed by Tukey’s post hoc test). *P < 0.05, **P < 0.01, ***P < 0.001.

To delineate the mechanistic basis of cholesterol-induced ferroptosis, we investigated the roles of cholesterol and PPARδ signaling in hepatocytes. C11-BODIPY staining revealed that cholesterol exposure significantly elevated lipid peroxidation in both AML12 and HepG2 cells, an effect that was effectively reversed by the ferroptosis inhibitor Fer-1 (Fig. 2a,b). To confirm the specificity of this cell death pathway, we tested various cell death inhibitors and found that only Fer-1 (not apoptosis (z-VAD), necrosis (IM-54) or necroptosis (z-VAD-fmk) inhibitors), rescued hepatocytes from cholesterol-induced death, supporting a ferroptotic mechanism (Supplementary Fig. 2a,b). In addition, cholesterol failed to induce ferroptosis in HSCs (LX2), indicating a hepatocyte-specific susceptibility (Supplementary Fig. 2c). Consistent with our prior in vivo findings implicating PPARδ in regulation of ferroptosis, cholesterol treatment selectively downregulated expression of PPARδ in hepatocytes at both the mRNA and protein level, whereas the expression of PPARα and PPARγ remained unchanged (Fig. 2c,d). Importantly, this reduction in PPARδ was driven by a dual mechanism involving both the suppression of de novo mRNA synthesis and rapid mRNA decay, as demonstrated by dual-luciferase reporter and actinomycin D chase assays (Supplementary Fig 2d,e). This phenomenon is reminiscent of previously described mechanisms where high cholesterol induces the destabilization of metabolic transcripts^40,41^. Given the established role of PPARδ in metabolic and redox control, we next examined whether its suppression modulates ferroptosis through antioxidant defense pathways. Notably, both xCT (a cystine–glutamate antiporter) and GPX4 were downregulated significantly at the transcript and protein level following cholesterol exposure, accompanied by a marked reduction in intracellular GSH levels (Fig. 2e–h). These changes suggest that impaired cystine uptake and GSH synthesis underlie the compromised ferroptosis defense in cholesterol-treated hepatocytes. To further establish the role of PPARδ, we silenced its expression in AML12 and HepG2 cells, which recapitulated the effects of cholesterol by enhancing lipid peroxidation and downregulating expression of xCT and GPX4 (Fig. 2i,j and Supplementary Fig. 2f). Of note, immunoblotting revealed no activation of cleaved caspase-3 following knockdown of PPARδ (Supplementary Fig. 2g), indicating that the observed cell death is not mediated by apoptosis. Taken together, these findings identify PPARδ as a central regulator of cholesterol-induced ferroptosis in hepatocytes, acting through suppression of the xCT–GPX4 antioxidant axis.

### Activation of PPARδ mitigates cholesterol-induced ferroptosis in hepatocytes via transcriptional upregulation of xCT

**Fig. 3:**
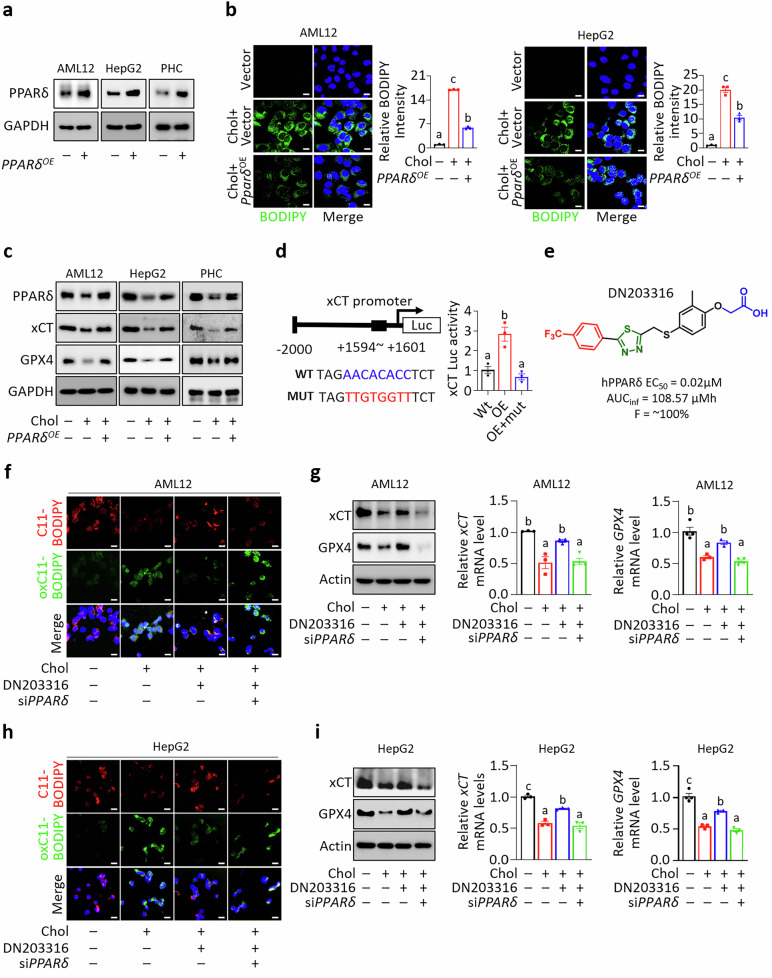
Activation of PPARδ attenuates cholesterol-induced ferroptosis in hepatocytes. **a** An immunoblot analysis of PPARδ levels in hepatocytes transfected with or without the overexpression (OE) vectors. **b** Representative BODIPY-stained images showing lipid peroxidation in cholesterol-treated hepatocytes with or without PPARδ OE. The quantification of BODIPY-positive cells is shown. **c** Levels of PPARδ, xCT and GPX4 proteins in cholesterol-treated hepatocytes with or without PPARδ OE. **d** A schematic diagram of luciferase reporter constructs containing full-length or mutant xCT promoter regions and luciferase activity in the presence or absence of PPARδ OE. **e** The chemical structure of DN203316, a selective PPARδ agonist. **f**,**h** The effect of DN203316 on lipid peroxidation in cholesterol-treated AML12 (**f**) or HepG2 (**h**) cells with or without PPARδ silencing. **g**,**i** The expression levels of xCT and GPX4 protein and mRNA in cholesterol-treated AML12 (**g**) or HepG2 (**i**) cells with or without PPARδ silencing. Data are expressed as the mean ± s.e.m. of three independent experiments. Samples denoted by different letters (**a**, **b** and **c**) vary significantly (*P* < 0.05, one-way ANOVA followed by Tukey’s post hoc test). n.s., not significant. *P < 0.05, **P < 0.01, ***P < 0.001.

To determine whether overexpression of PPARδ confers protection against cholesterol-induced ferroptosis, we generated stable hepatocyte cell lines overexpressing PPARδ via lentiviral transduction, which led to a marked increase in PPARδ protein levels compared with vector controls (Fig. 3a and Supplementary Fig. 3a). The overexpression of PPARδ attenuated cholesterol-induced lipid peroxidation significantly in both AML12 and HepG2 cells (Fig. 3b). This protective effect was accompanied by restored expression of xCT and GPX4, two key ferroptosis-defense genes previously shown to be suppressed by cholesterol (Fig. 3c). Consistent with these changes, cells overexpressing PPARδ exhibited elevated intracellular cysteine and GSH levels, suggesting the enhancement of antioxidant capacity via the cystine–GSH axis (Supplementary Fig. 3b,c). In contrast to this xCT-dependent protection, the expression of canonical ferroptosis regulators, including ACSL4, LPCAT3 and ALOX15, remained unaffected by either cholesterol stimulation or PPARδ agonist treatment. Moreover, although SLC3A2—the subunit that heterodimerizes with xCT in the cystine–glutamate antiporter—was downregulated by cholesterol, its expression was not rescued by the PPARδ agonist treatment (Supplementary Fig. 3d). To determine whether PPARδ regulates xCT transcription directly, we conducted luciferase reporter assays using wild-type and mutant xCT promoter constructs. The overexpression of PPARδ led to a robust increase in xCT promoter activity, whereas the mutation of the putative PPARδ-binding motif abolished this effect, confirming transcriptional regulation of xCT by PPARδ (Fig. 3d). Building on these findings, we next investigated whether pharmacological activation of PPARδ could phenocopy the protective effects of PPARδ overexpression. Treatment with DN203316, a selective PPARδ agonist developed in our previous study (Fig. 3e), suppressed cholesterol-induced hepatocyte death significantly without detectable toxicity (Supplementary Fig. 3e,f). DN203316 also reduced lipid peroxidation in AML12 and HepG2 cells, an effect that was abrogated by knockdown of PPARδ (Fig. 3f,h and Supplementary Fig. 3g). Mechanistically, DN203316 restored expression of xCT and GPX4 at both the transcript and protein levels and increased GSH levels in cholesterol-exposed hepatocytes, all of which were dependent on PPARδ, as knockdown abolished these responses (Fig. 3g,i and Supplementary Fig. 3h). Taken together, these findings demonstrate that both the genetic and pharmacologic activation of PPARδ mitigates cholesterol-induced ferroptosis in hepatocytes through the transcriptional induction of xCT, positioning PPARδ as a promising therapeutic target in ferroptosis-driven liver injury during MASH progression.

### Ferroptotic hepatocyte-derived exosomal dsDNA activates STING signaling in HSCs and promotes fibrogenesis in MASH

**Fig. 4:**
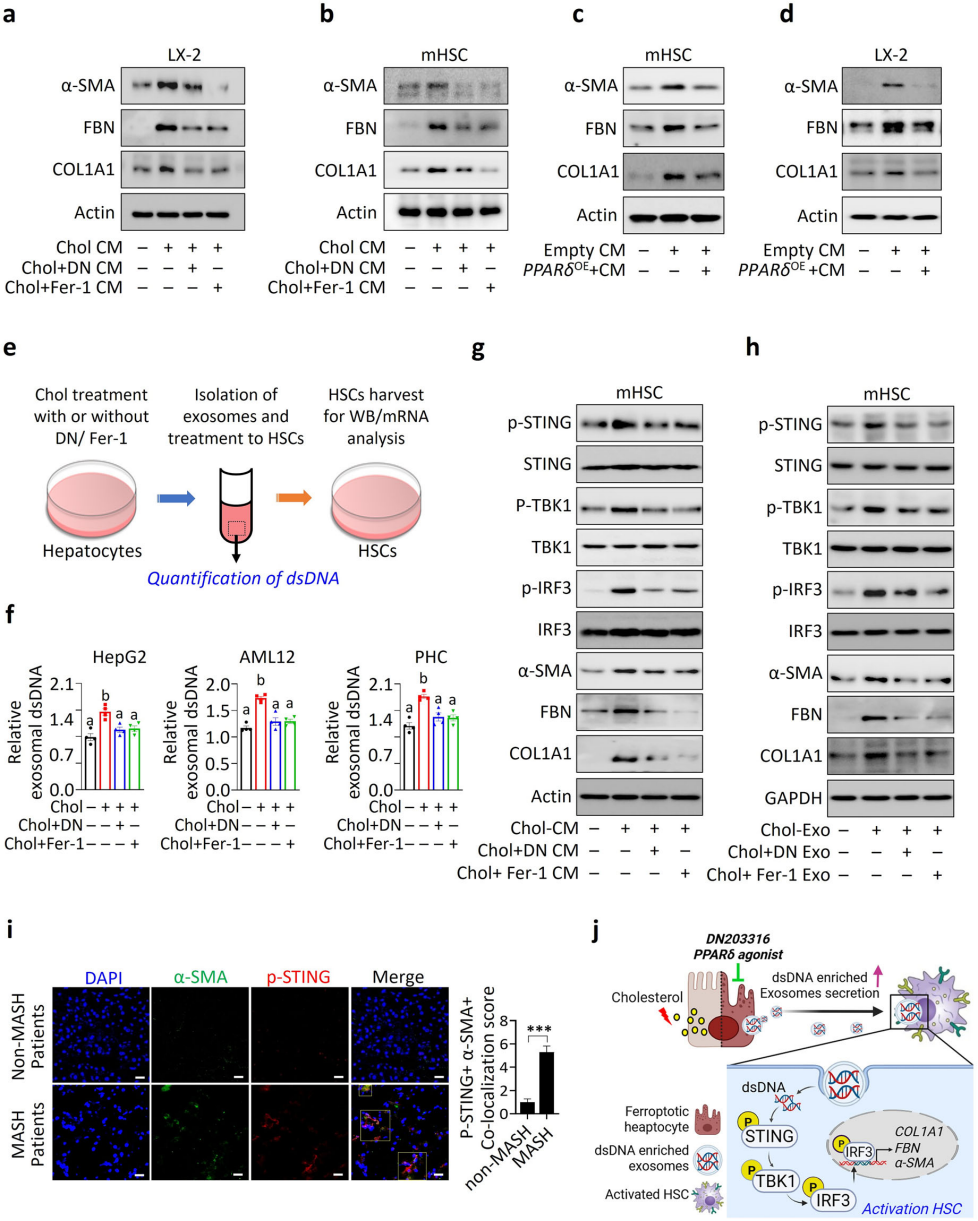
Activation of PPARδ reduces exosomal dsDNA release from ferroptotic hepatocytes and inhibits STING-driven fibrogenesis in MASH. **a**,**b** Levels of α-SMA, fibronectin (FBN) and COL1A1 protein in human (**a**) and mouse HSCs (**b**) incubated with CM (conditioned media) from cholesterol-treated hepatocytes with or without DN203316 or Fer-1. **c**,**d** Levels of α-SMA, FBN and COL1A1 protein in mouse (**c**) and human HSCs (**d**) incubated with CM from cholesterol-treated hepatocytes transfected with PPARδ OE vectors. **e** A schematic diagram of the experimental protocol for dsDNA isolation and HSC stimulation. **f** The quantification of extracellular dsDNA in CM from cholesterol-treated hepatocytes with or without DN203316 or Fer-1. **g**,**h** The phosphorylation of STING, TBK1 and IRF3 and expression of fibrogenic markers in mouse primary HSCs treated with CM (**g**) or isolated exosomes (**h**) from cholesterol-treated hepatocytes with or without DN203316 or Fer-1. **i** Left: representative immunofluorescence images showing the colocalization of α-SMA and phosphorylated STING in liver sections from patients with MASH. Right: the quantification of double-positive cells is shown. **j** A schematic model depicting the proposed mechanism by which the PPARδ agonist DN203316 attenuates hepatocyte ferroptosis and suppresses HSC activation via the inhibition of STING–TBK1–IRF3 signaling. Data are expressed as the mean ± s.e.m. of three independent experiments. Scale bar, 60 µm. Samples denoted by different letters (**a** and **b**) vary significantly (*P* < 0.05, one-way ANOVA followed by Tukey’s post hoc test). *P < 0.05, **P < 0.01.

Although our in vivo findings demonstrated fibrosis following a CED, the direct induction of fibrotic genes in HSCs by cholesterol alone was minimal compared with TGF-β1 (Supplementary Fig. 4a,b). This discrepancy prompted us to investigate whether cholesterol drives HSC activation indirectly via hepatocyte-derived mediators, particularly those released during ferroptosis. To this end, we exposed HSCs to CM from cholesterol-treated hepatocytes and measured production of extracellular matrix. Indeed, CM from both human and mouse hepatocytes increased α-SMA, fibronectin and COL1A1 expression robustly in HSCs, whereas CM derived from hepatocytes cotreated with the PPARδ agonist DN203316 or the ferroptosis inhibitor Fer-1 did not (Fig. 4a,b). Similarly, CM from PPARδ-overexpressing hepatocytes, which are protected from ferroptosis, did not induce expression of fibrotic markers in HSCs (Fig. 4c,d). Emerging studies implicate hepatocyte-derived extracellular vesicles (EVs), including exosomes, as key paracrine mediators in MASH through the delivery of bioactive molecules such as proteins, lipids and dsDNA. Therefore, we isolated exosomes from cholesterol-treated hepatocytes and quantified their total dsDNA content, which included a substantial mtDNA fraction. This DNA cargo increased markedly in a ferroptosis-dependent manner and was suppressed by DN203316 or Fer-1, indicating that exosomal dsDNA enrichment is a downstream consequence of hepatocyte ferroptosis (Fig. 4e,f and Supplementary Fig. 4c). As STING–TBK1–IRF3 signaling plays a pivotal role in HSC activation and fibrogenesis, we next asked whether dsDNA-enriched exosomes from ferroptotic hepatocytes activate this pathway in HSCs. CM and exosomes derived from cholesterol-treated hepatocytes strongly induced phosphorylation of STING, TBK1 and IRF3, as well as upregulation of fibrotic markers in HSCs. These effects were abolished when exosomes originated from hepatocytes treated with DN203316 or Fer-1 (Fig. 4g,h and Supplementary Fig. 4d). Importantly, DNase I digestion, which degrades exosomal dsDNA, markedly suppressed STING–TBK1–IRF3 signaling, confirming the functional contribution of exosomal dsDNA in HSC activation (Supplementary Fig. 4e). Notably, direct treatment with cholesterol did not activate the STING pathway in either hepatocytes or HSCs (Supplementary Fig. 4f,g), reinforcing the importance of hepatocyte-derived exosomal signaling in mediating HSC activation. To further confirm the role of EVs, we silenced Rab27a to inhibit exosome release, which resulted in CM from cholesterol-treated hepatocytes failing to activate STING signaling or expression of fibrotic markers in HSCs (Supplementary Fig. 4h,i). Moreover, the pharmacologic inhibition of STING with SN-011 suppressed downstream signaling and fibrotic responses in HSCs exposed to CM from cholesterol-treated hepatocytes (Supplementary Fig. 4j). Beyond direct fibrogenesis, hepatocyte ferroptosis further shapes the inflammatory microenvironment in MASH. In flow cytometry-verified primary KCs (defined as F4/80^high^ CD11b^int^) (Supplementary Fig. 5a), CM from ferroptotic hepatocytes potently induced the secretion of TNF-α, IL-6 and IL-1β, which was markedly attenuated by DN203316 (Supplementary Fig. 5b). In parallel, CM from cholesterol-treated hepatocytes significantly upregulated the expression of IL-6 and CXCL12 in both human and mouse primary HSCs, establishing a proinflammatory and profibrogenic axis that was effectively blocked by PPARδ activation (Supplementary Fig. 5c–f). These findings revealed an inflammatory–fibrogenic cascade linking hepatocyte ferroptosis to KC activation and HSC transdifferentiation. Finally, immunofluorescence analysis of human liver samples revealed a significant increase in p-STING/α-SMA double-positive cells in MASH tissues, underscoring the clinical relevance of STING-mediated activation of HSCs (Fig. 4i). Collectively, these findings identify exosomal dsDNA released from ferroptotic hepatocytes as a key upstream trigger of STING–TBK1–IRF3 activation in HSCs, linking cholesterol-induced ferroptosis of hepatocytes to HSC-mediated fibrogenesis in MASH and positioning PPARδ activation as a potential therapeutic intervention (Fig. 4j).

### Hepatic overexpression of PPARδ attenuates MASH progression by suppressing ferroptosis and fibrogenesis

**Fig. 5:**
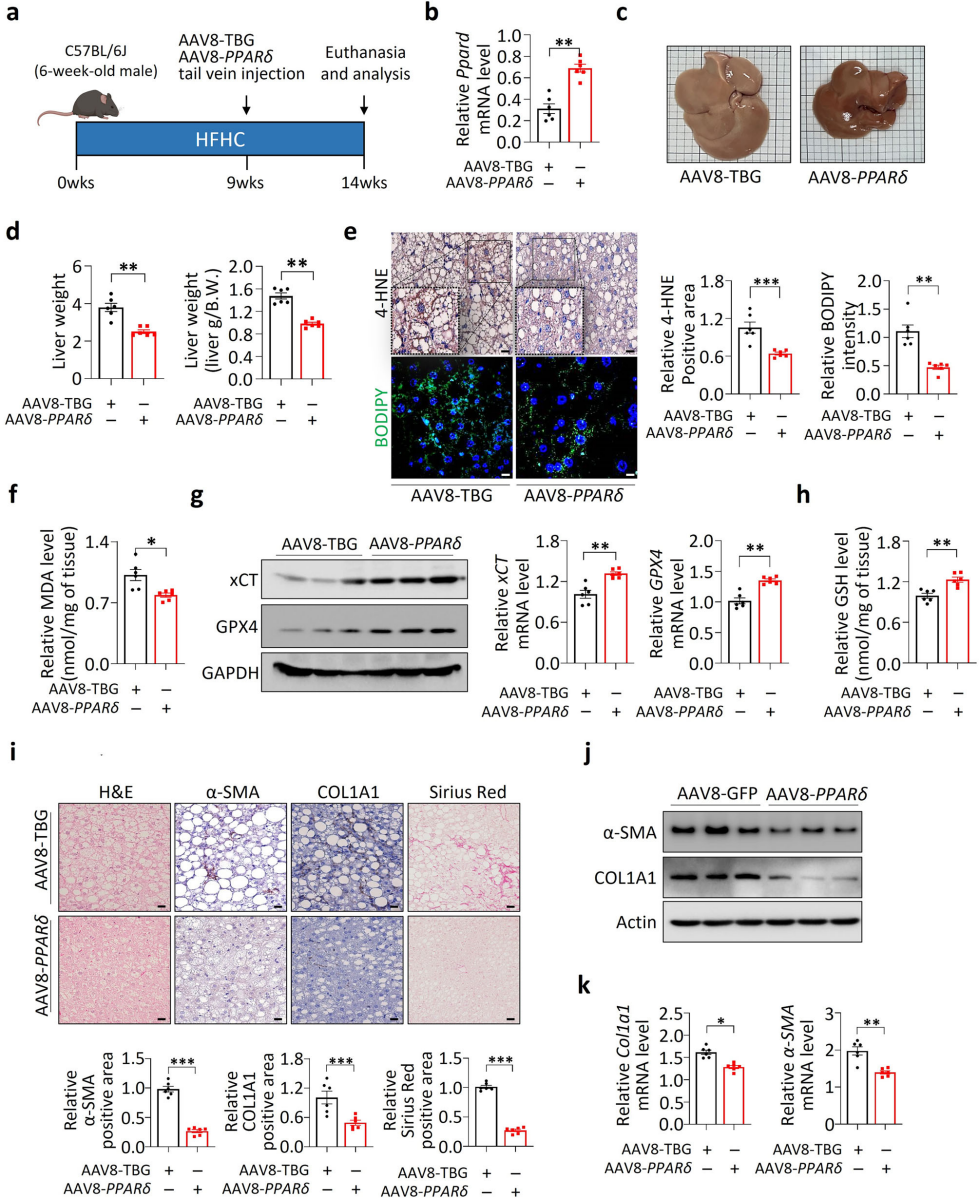
Overexpression of PPARδ attenuates liver fibrosis by mitigating ferroptosis in an HFHC diet-induced MASH model. **a** A schematic overview of the HFHC-AAV8-TBG-PPARδ model. C57BL/6J mice were fed a HFHC diet for 9 weeks, followed by weekly intravenous injections of AAV8-TBG-PPARδ, with analysis performed at week 14 (*n* = 6 per group). **b**–**d** The hepatic expression of PPARδ mRNA (**b**), gross liver morphology (**c**) and liver weight (**d**) in AAV8-TBG-PPARδ-treated HFHC-fed mice. **e** Left: representative images of BODIPY staining and IHC analysis of 4-HNE in liver sections of AAV8-PPARδ-treated MASH mice. Right: the quantification of positive cells. **f**–**h** The hepatic MDA levels (**f**), xCT and GPX4 expression at the protein and mRNA levels (**g**) and hepatic GSH content (**h**) in HFHC-fed mice with or without AAV8-TBG-PPARδ overexpression. **i** Top: representative histological images (H&E), IHC analysis of α-SMA and COL1A1 and Sirius red staining of liver sections. Bottom: the quantification of fibrosis-related positive areas is shown. **j**,**k** The expression of xCT and GPX4 protein (**j**) and mRNA (**k**) in AAV8-TBG-PPARδ-treated HFHC-fed mice. Data are expressed as the mean ± s.e.m. of three independent experiments. Scale bar, 20 µm. *P < 0.05, **P < 0.01, ***P < 0.001.

To assess the in vivo role of PPARδ in modulating MASH progression, we generated a liver-specific overexpression model using AAV8 carrying the hepatocyte-specific TBG promoter (AAV8-TBG-PPARδ), with AAV8-TBG-GFP as a control (Fig. 5a,b). To maintain robust expression during the chronic fibrogenic phase, mice received five weekly injections starting at week 9. Importantly, this repeated dosing regimen did not provoke significant hepatic inflammation (TNF-α, IL-6 and IL-1β) or a neutralizing anti-AAV8 IgG response, ensuring efficient and safe gene transfer (Supplementary Fig. 6a,b). After 14 weeks of HFHC feeding, AAV8-TBG-PPARδ-treated mice exhibited a marked improvement in liver morphology, with reduced pallor and swelling (Fig. 5c). In addition, we noted a significant decrease in liver weight and serum ALT/AST levels, with no effects on body weight, fat mass, food intake or induction of liver toxicity (Fig. 5d and Supplementary Fig. 6c–f). Lipid peroxidation was suppressed strongly in PPARδ-overexpressing livers, as shown by decreased 4-HNE staining, reduced C11-BODIPY fluorescence and lower malondialdehyde (MDA) levels (Fig. 5e,f). In line with these observations, antioxidant capacity was restored, as evidenced by the increased expression of xCT and GPX4 and elevated expression of hepatic GSH (Fig. 5g,h). The AAV-PPARδ treatment markedly reduced hepatic fibrosis, as evidenced by decreased α-SMA immunoreactivity, COL1A1 expression and Sirius red staining, with these antifibrotic effects further confirmed by consistent downregulation of α-SMA and Col1a1 at both the protein and transcript levels (Fig. 5i–k). Collectively, these findings demonstrate that hepatocyte-specific overexpression of PPARδ suppresses ferroptosis and fibrogenesis in response to a cholesterol-rich diet, thereby attenuating MASH progression.

### DN203316 alleviates MASH by suppressing ferroptosis and STING-driven fibrogenesis in a MASH mouse model

**Fig. 6:**
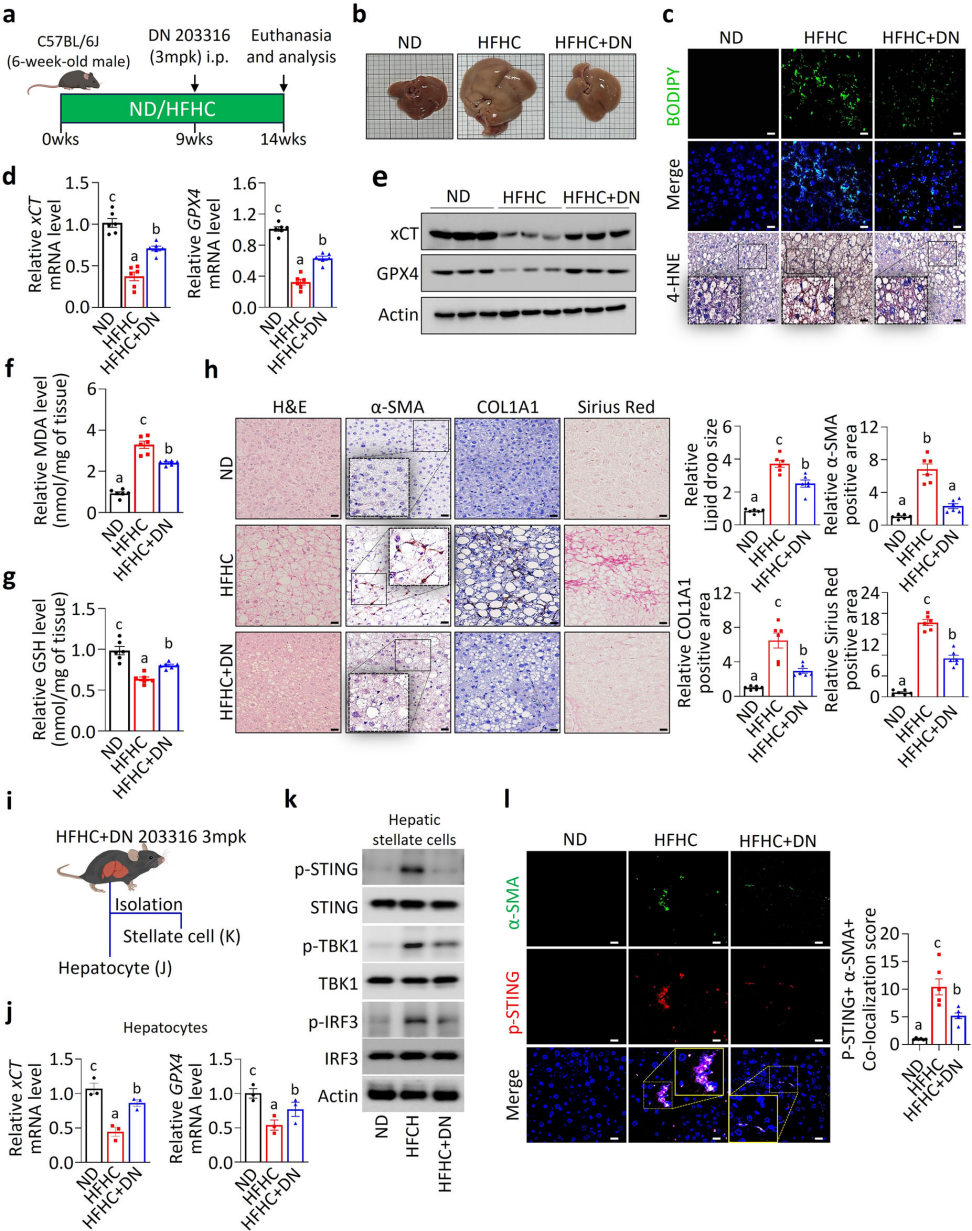
DN203316 ameliorates liver fibrosis by mitigating ferroptosis in an HFHC-induced MASH mouse model. **a** A schematic showing the DN203316 treatment protocol in HFHC-induced MASH mice (*n* = 6 per group). **b** The representative images of liver morphology in HFHC-fed mice treated with DN203316. **c**–**g** The assessment of hepatic ferroptosis markers in HFHC-fed mice treated with DN203316, including lipid peroxidation (BODIPY staining) (**c**, top), IHC analysis of 4-HNE (**c**, bottom), xCT and GPX4 mRNA (**d**) and protein levels (**e**) and hepatic MDA (**f**) and GSH levels (**g**). **h** Left: the histological evaluation of liver fibrosis using H&E, IHC analysis of α-SMA and COL1A1 and Sirius red staining. Right: the quantification of positive areas. **i**–**k** A schematic showing the isolation of hepatocytes and HSCs from HFHC-fed mice treated with DN203316 (3 mg/kg, *n* = 3 per group) (**i**); xCT and GPX4 mRNA levels were measured in primary hepatocytes (**j**), whereas protein levels of phosphorylated STING (p-STING), TBK1 (p-TBK1) and IRF3 (p-IRF3) were assessed in primary HSCs (**k**). **l** Left: representative immunofluorescence images showing colocalization of α-SMA and p-STING in liver sections of DN203316-treated MASH mice. Right: the quantification of double-positive cells. Data are expressed as the mean ± s.e.m. of three independent experiments. Scale bar, 20 µm; yellow scale bar, 60 µm. Samples denoted by different letters (**a**, **b** and **c**) vary significantly (*P* < 0.05, one-way ANOVA followed by Tukey’s post hoc test). *P < 0.05, **P < 0.01, ***P < 0.001.

To determine whether pharmacologic activation of PPARδ recapitulates the antifibrotic and anti-ferroptotic effects associated with overexpression of PPARδ, we administered the selective PPARδ agonist DN203316 (3 mg/kg/day, i.p.) to HFHC-fed mice for 5 weeks (Fig. 6a). DN203316 improved liver morphology, including reduced liver weight, serum ALT/AST levels, markedly without altering body weight, adiposity, or food intake (Fig. 6b and Supplementary Fig. 7a–e). Hepatic ferroptosis induced by the HFHC diet was attenuated significantly by DN203316, as indicated by reduced accumulation of 4-HNE, diminished BODIPY fluorescence, decreased hepatic MDA levels and restoration of xCT and GPX4 expression alongside increased hepatic GSH content (Fig. 6c–g and Supplementary Fig. 7f), thereby recapitulating the redox recovery observed after the overexpression of PPARδ. Histopathological analyses further revealed that DN203316 caused a marked reduction in hepatic fibrosis, as shown by diminished Sirius red staining and decreased expression of α-SMA and Col1a1 at both the mRNA and protein levels (Fig. 6h and Supplementary Fig. 7g,h). To dissect the underlying cellular mechanisms, we isolated primary hepatocytes and HSCs from HFHC-fed mice treated with DN203316 (Fig. 6i). In isolated primary hepatocytes, DN203316 restored expression of antioxidant genes, including xCT and GPX4 (Fig. 6j). In parallel, DN203316 inhibited activation of the STING–TBK1–IRF3 signaling axis in primary HSCs, a pathway that mediates ferroptosis-induced fibrogenesis (Fig. 6k). Immunofluorescence analysis confirmed a marked reduction in p-STING and α-SMA colocalization in liver tissues from DN203316-treated mice (Fig. 6l), indicating that DN203316 disrupts hepatocyte-to-HSC signaling during ferroptosis-driven fibrogenesis in MASH. Taken together, these findings demonstrate that the pharmacologic activation of PPARδ by DN203316 effectively suppresses ferroptosis and STING-driven fibrogenesis in MASH, providing a strong rationale for its development as a therapeutic agent for MASH.

## Discussion

Here, we show that cholesterol-induced ferroptosis of hepatocytes is a central pathogenic mechanism that drives MASH progression via the downregulation of PPARδ. Using a dietary model that recapitulates the pathophysiology of human MASH, we identified the PPARδ–xCT–GPX4 axis as a critical regulator of redox homeostasis and showed that genetic or pharmacological activation of PPARδ (via DN203316) mitigates disease progression. Notably, DN203316 suppressed the exosomal release of dsDNA from ferroptotic hepatocytes, thereby disrupting the activation of the STING pathway in HSC. These findings highlight a previously unrecognized axis linking metabolic stress to fibrogenic signaling and provide mechanistic insights into MASH-associated stromal remodeling.

Although ferroptosis has gained attention as a therapeutic target in chronic liver diseases, its upstream regulation in the context of MASH remains poorly understood. Our study demonstrates that cholesterol loading induces ferroptotic cell death in hepatocytes, which is inhibited selectively by Fer-1 but not by apoptosis or necroptosis inhibitors, confirming ferroptosis as the dominant mode of hepatocyte injury in cholesterol-induced MASH. We identified the selective downregulation of PPARδ in both cholesterol-treated hepatocytes and MASH liver tissues, suggesting that PPARδ is uniquely vulnerable to metabolic insult. Although cholesterol reduced PPARδ mRNA levels through effects on both transcriptional activity and mRNA stability, the magnitude of mRNA suppression was more modest than the robust decrease in protein abundance. This discrepancy implies that additional layers of regulation, including altered translational efficiency and/or increased protein turnover, probably further contribute to cholesterol‑induced PPARδ downregulation, representing compelling areas for future investigation. The resulting deficiency in PPARδ signaling subsequently impaired transcriptional regulation of the xCT–GPX4 antioxidant axis, leading to redox imbalance and increased susceptibility to ferroptosis. Although classical PPARδ agonists such as GW501516 and GW0742 are limited by concerns over lack of evidence for clinical effectiveness in MASH in rodent models, these compounds have proved invaluable for elucidating the diverse roles of PPARδ in metabolic and inflammatory regulation, thereby underscoring its therapeutic potential^42^. To advance this pharmacological framework, we developed DN203316, a highly potent and selective PPARδ agonist featuring a 1,3,4-thiadiazole scaffold with an excellent drug-likeness profile that confers more than 400-fold selectivity against PPARα and PPARγ. This structural specificity probably prevents the activation of off-target pathways implicated in the carcinogenicity of earlier agonists. In the present study, both genetic overexpression and pharmacologic activation of PPARδ with DN203316 effectively protected against cholesterol-induced ferroptosis and attenuated MASH progression in vitro and in vivo, without detectable toxicity. These findings position DN203316 as a mechanistically informed and selectively potent therapeutic candidate for ferroptosis-driven liver injury, offering a safer alternative to earlier PPARδ agonists for treatment of MASH.

The dynamic interplay between hepatocytes and nonparenchymal cells constitutes a critical multicellular network driving the pathogenesis of liver fibrosis^43–46^. Our data reveal that one major downstream consequence of hepatocyte ferroptosis is release of EVs, particularly exosomes enriched in dsDNA, which function as paracrine mediators facilitating hepatocyte–HSC communication. These exosomes, which may carry iron, lipid peroxidation byproducts and nucleic acids appear to play a central role in promoting fibrogenic signaling within the MASH microenvironment^47,48^. Consistent with growing evidence implicating EVs in the pathogenesis of liver fibrosis^49–51^, we demonstrate that cholesterol-induced hepatocyte ferroptosis increases the release of dsDNA-containing exosomes significantly. Notably, the inhibition of ferroptosis by restoration of PPARδ signaling via DN203316 mitigated both the production and fibrogenic potential of these vesicles, underscoring ferroptosis as a central determinant of vesicle cargo and intercellular communication. These observations suggest a model in which ferroptotic hepatocytes act not simply as damaged cells but as active contributors to the stromal niche, linking metabolic stress and redox imbalance to paracrine fibrotic signaling in MASH.

Building on this framework, we identified the STING–TBK1–IRF3 signaling axis as a critical downstream effector of dsDNA-containing exosomes that promote HSC activation. Mechanistically, STING activation—probably triggered predominantly by mtDNA within the exosomal dsDNA cargo—is known to drive hepatic inflammation and fibrosis in metabolic liver disease models such as metabolic dysfunction-associated steatotic liver disease/MASH. Recent evidence shows that ferroptosis is linked to substantial mitochondrial lipid peroxidation and damage, which promotes the release of hypomethylated mtDNA, a strong ligand for the cGAS–STING pathway. By contrast, nuclear DNA remains largely sequestered within the chromatin architecture and is not as readily exposed as cytosolic duplex DNA under these conditions^52–54^. Consistent with this model, we detected a substantial mtDNA fraction within hepatocyte‑derived exosomal dsDNA and found that these exosomes robustly activated STING signaling and induced fibrogenic genes such as α‑SMA and Col1a1 in HSCs—a response attenuated by interventions that suppress ferroptosis or restore PPARδ activity. These findings, together with prior work showing that stress‑induced mtDNA release can activate STING and promote collagen deposition in HSCs, support the concept that mtDNA‑enriched exosomal dsDNA represents a key molecular driver of STING‑dependent fibrogenesis in MASH^55^. The therapeutic relevance of this axis is further underscored by human MASH data revealing p-STING/α-SMA colocalization, which aligns with our mechanistic insights and highlights the clinical relevance of this pathway in fibrotic progression.

This study identifies cholesterol-induced ferroptosis as a key driver of MASH progression and establishes PPARδ as a central regulator of ferroptotic susceptibility. DN203316, a novel and highly selective PPARδ agonist, restored redox balance, suppressed the release of hepatocyte-derived dsDNA-containing exosomes and attenuated STING-mediated fibrogenesis. These findings delineate a mechanistic link between metabolic stress, ferroptotic signaling and stromal activation, positioning DN203316 as a promising candidate for clinical trials of treatments for metabolic liver disease.

## Supplementary information

Supplementary Information

## Data Availability

Further information and requests for resources and reagents should be directed to and will be fulfilled by the lead contact, K.-G.P. (kpark@knu.ac.kr).
