# Supplementary material for: DN203316, a novel PPARδ agonist, suppresses ferroptotic signaling and fibrogenesis in metabolic dysfunction-associated steatohepatitis
Source: Exp Mol Med. 2026 Jun 5;58(6):1900–13. doi: 10.1038/s12276-026-01740-0 (PMC13324369; doi:10.1038/s12276-026-01740-0)
Supplement: Supplementary file 1 — Supplementary Information [file 12276_2026_1740_MOESM1_ESM.pdf]

DN203316, a novel PPAR δ Agonist, Suppresses Ferroptotic Signaling and Fibrogenesis in Metabolic Dysfunction-associated Steatohepatitis

Ye Jin Kim[1,2]*, Jina Kim[3]*, Da Young An[4], Mihyang Park[1,2], Gui-Hwa Jeong[5], Jonghwa Jin[1,2], Mi Kyung Kim[6], Jungwook Chin[7**], Yeon-Kyung Choi[2,8**], Keun-Gyu Park[1,2,4**]

[1]Department of Internal Medicine, School of Medicine, Kyungpook National University, Kyungpook National University Hospital, Daegu 41944, South Korea
[2]Research Institute of Aging and Metabolism, Kyungpook National University, Daegu 41566, Republic of Korea
[3]New Drug Development Center, Daegu-Gyeongbuk Medical Innovation Foundation, Daegu, 41061, Republic of Korea
[4]Department of Biomedical Science, Kyungpook National University, Daegu 41566, Republic of Korea
[5]Departmentof Internal Medicine, CHA Gumi Medical Center, CHA University, Gumi 39295, Republic of Korea
[6]Department of Internal Medicine, Keimyung University School of Medicine, Daegu 42601, South Korea
[7]Cureverse, lnc., V1 building, KIST, Seoul 02792, Republic of Korea
[8]Department of Internal Medicine, School of Medicine, Kyungpook National University, Kyungpook National University Chilgok Hospital, Daegu 41404, South Korea

Supplementary Fig. 1

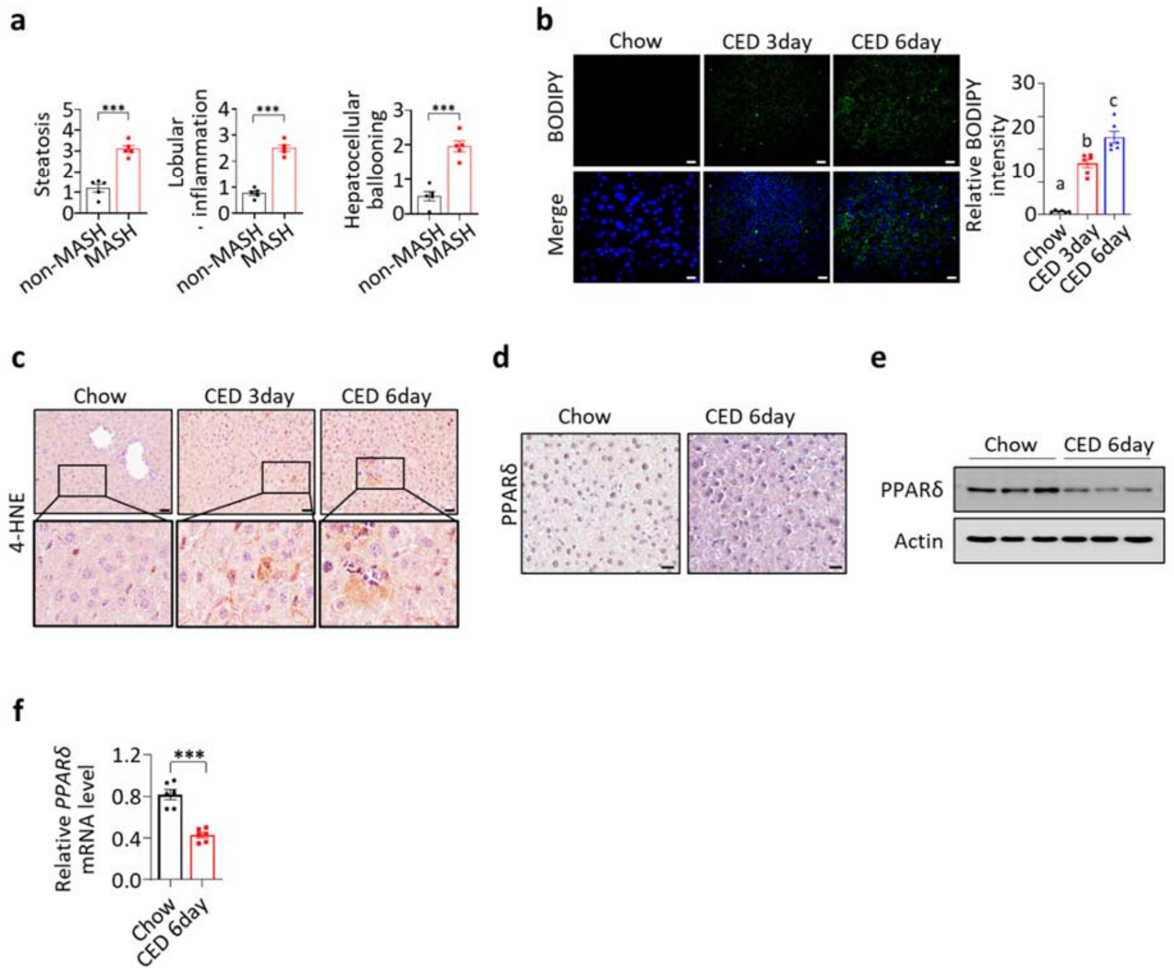

Supplementary Fig. 1. Cholesterol-enriched diet induces hepatic ferroptosis and downregulates expression of PPARδ. (a) Quantification of pathological scores in liver tissues from non-MASH and MASH patients. (b-f) Liver tissues from mice fed a cholesterol-enriched diet (CED) were analyzed by immunofluorescence staining with BODIPY to detect lipid peroxidation (b), IHC analysis to detect 4-HNE (c) and PPARδ (d), and by immunoblotting and quantitative PCR to detect hepatic PPARδ protein (e) and mRNA (f). Data are expressed as the mean ± SEM of three independent experiments. Scale bar, 20 μm. Samples denoted by different letters (a, b and c) vary significantly ($p < 0.05$, one-way ANOVA followed by Tukey's post hoc test). *$p < 0.05$ and ***$p < 0.001$.

Supplementary Fig. 2

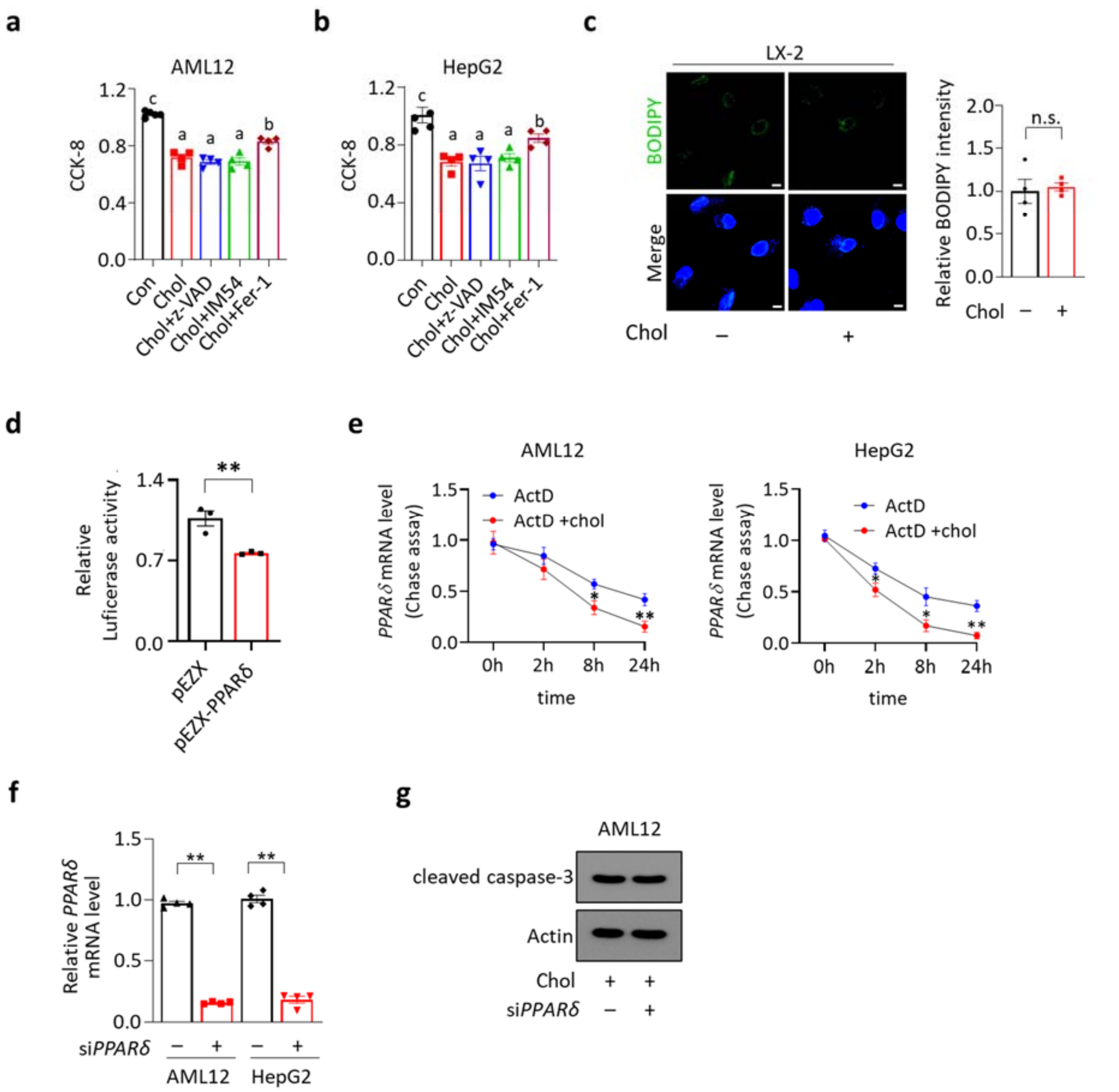

Supplementary Fig. 2. Cholesterol induces ferroptosis, but not apoptosis, in hepatocytes. (a, b) Viability of cholesterol-treated AML12 (a) and HepG2 (b) cells in the presence or absence of inhibitors targeting necrosis (IM-54), apoptosis (z-VAD-FMK), and ferroptosis (ferrostatin-1). (c) Representative immunofluorescence images of lipid peroxidation, as detected by BODIPY staining in LX-2 cells following exposure to cholesterol (left panel). Quantification of BODIPY-positive cells is shown (right panel). (d, e) PPARδ dual-luciferase reporter assay and actinomycin D chase assay in hepatocytes treated with cholesterol. (f) Confirmation of

PPARδ knockdown in AML12 and HepG2 cells. (g) Immunoblot analysis of cleaved caspase-3 in cholesterol-treated AML12 cells under PPARδ-silenced conditions. Data are expressed as the mean ± SEM of three independent experiments. Scale bar, 20 μm. Samples denoted by different letters (a and b) vary significantly ($p < 0.05$, one-way ANOVA followed by Tukey's post hoc test). n.s.; not significant. $^{**}p < 0.01$ and $^{***}p < 0.001$.

Supplementary Fig. 3

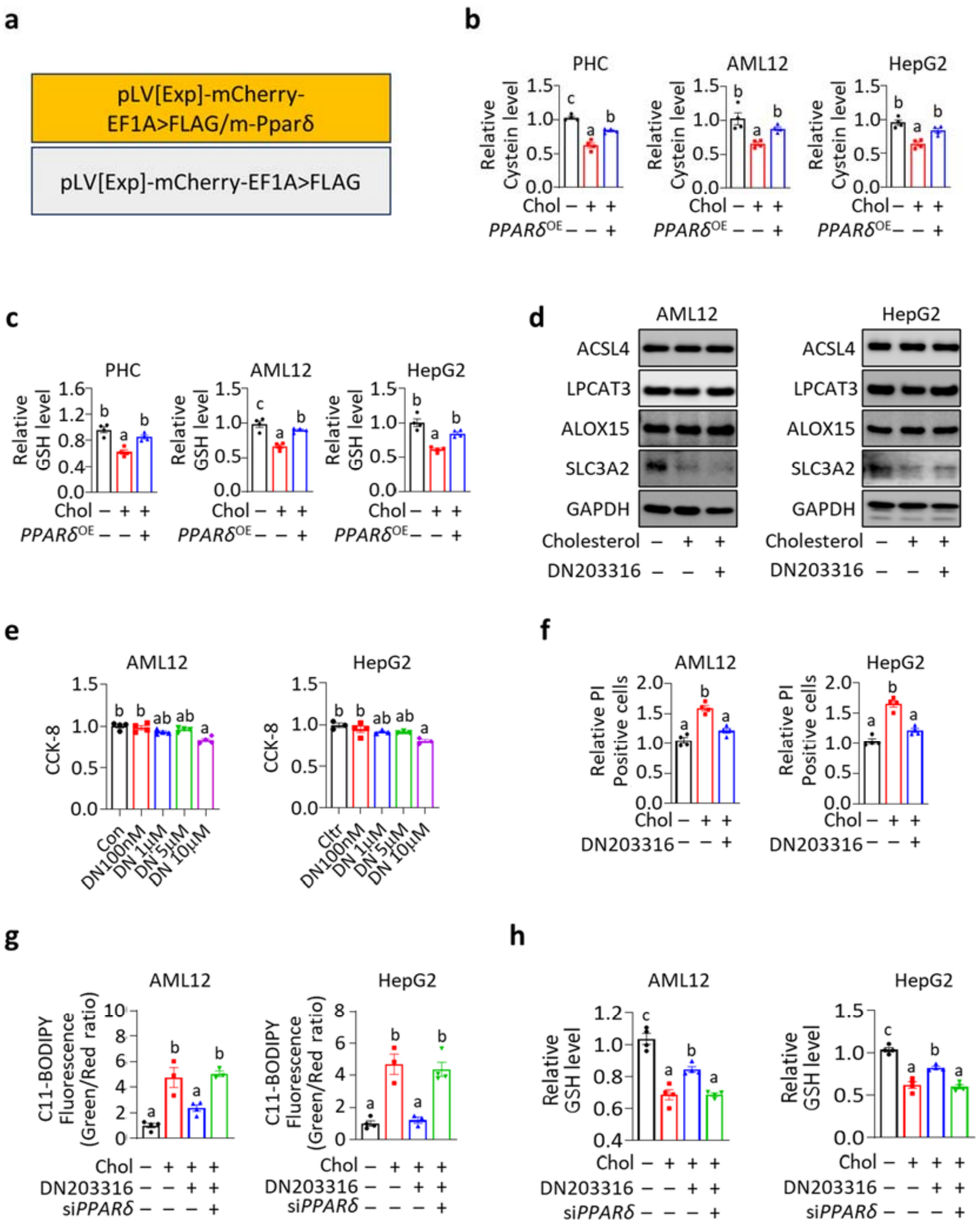

Supplementary Fig. 3. Effect of PPARδ overexpression or agonist DN203316 on hepatocyte ferroptosis. (a) Schematic representation of the luciferase reporter constructs containing either the full-length or a mutated xCT promoter, and luciferase activity in the presence or absence of PPARδ overexpression (OE). (b, c) Effects of PPARδ OE or DN203316

on intracellular cysteine (b) and GSH levels (c) in cholesterol-treated hepatocytes. (d) Protein levels of ACSL4, LPCAT3, ALOX15, and SLC3A2 in hepatocytes treated with cholesterol with or without DN203316. (e) Cytotoxicity of DN203316 in hepatocytes, as assessed in the CCK-8 assay. (f) Effects of DN203316 on viability in cholesterol-treated hepatocytes, as measured by propidium iodide (PI) staining. (g) Quantitative analysis of BODIPY staining shown in Fig. 3f and 3h. (h) Effects of DN203316 on GSH levels in cholesterol-treated hepatocytes with or without PPARδ silencing. Data are expressed as the mean ± SEM of three independent experiments. Samples denoted by different letters (a, b and c) vary significantly ($p < 0.05$, one-way ANOVA followed by Tukey's post hoc test). n.s.; not significant. *$p < 0.05$, **$p < 0.01$, and ***$p < 0.001$.

Supplementary Fig. 4

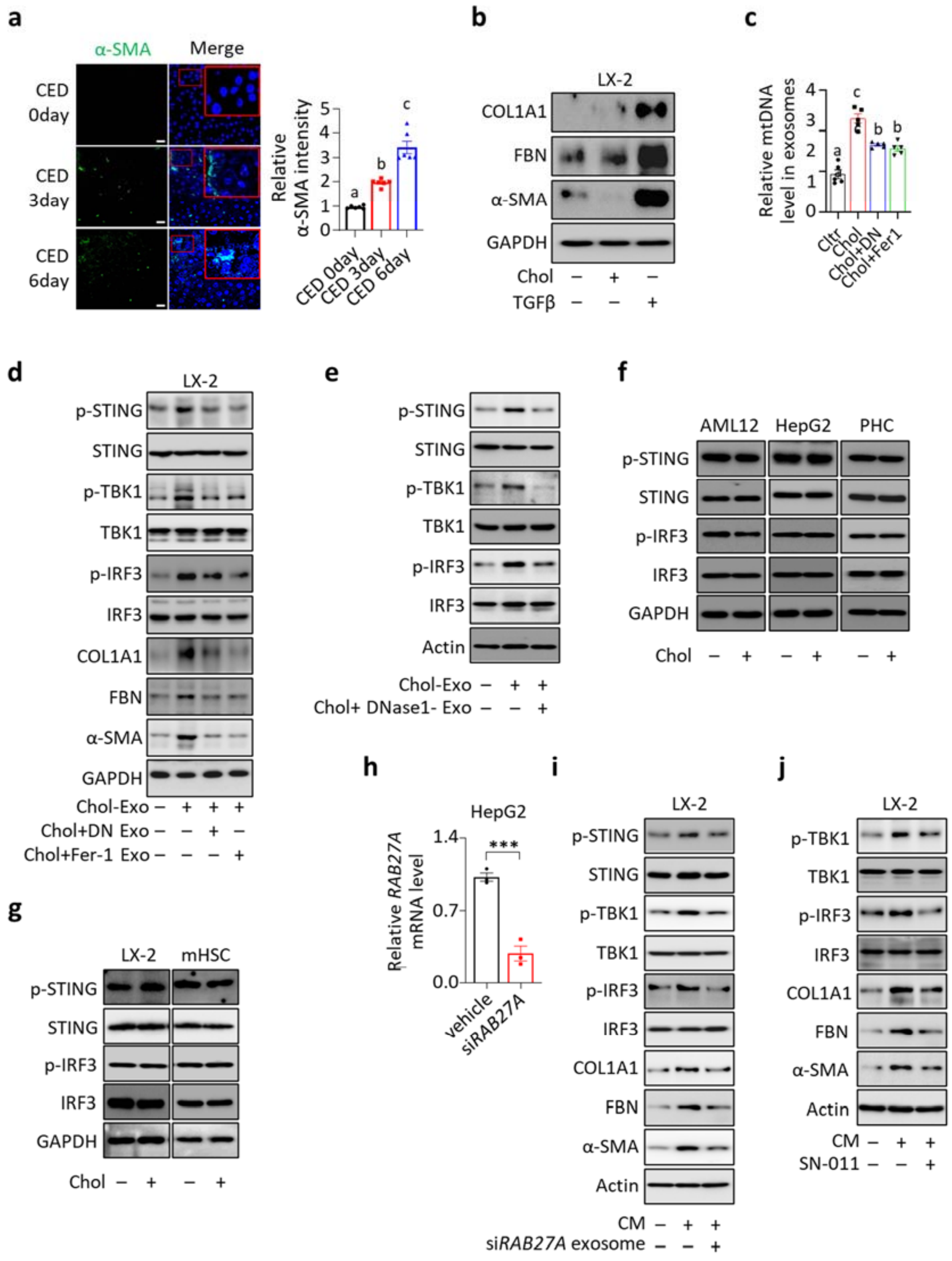

Supplementary Fig. 4. DN203316 and Exosome-Mediated Crosstalk Regulate HSC Activation via the STING/TBK1/IRF3 Pathway. (a) Representative immunofluorescence images showing α-SMA expression in liver tissues during cholesterol-enriched diet feeding as

shown in Supplementary Fig. 1a (left panel). Quantification of positive cells is shown (right panel). (b) Protein levels of fibrogenic markers in LX-2 cells treated with cholesterol or TGF-β. (c) Quantification of exosomal mtDNA, showing that the mitochondrial DNA fraction is enriched in exosomes derived from cholesterol-treated HepG2 cells. (d) Effects of exosomes derived from cholesterol-treated hepatocytes with or without DN203316 or ferrostatin-1 (Fer-1) on phosphorylation of STING, TBK1, and IRF3, and expression of fibrogenic markers in LX-2 cells. (e) Effects of exosomes derived from cholesterol-treated hepatocytes with or without DNase I on phosphorylation of STING, TBK1, and IRF3 in LX-2 cells. (f–g) Protein levels of phosphorylated STING and IRF3 in cholesterol-treated human and mouse hepatocytes (f) or hepatic stellate cells (g). (h) Validation of Rab27a knockdown efficiency in HepG2 cells. (i) Effects of conditioned media (CM) from cholesterol-treated HepG2 cells with or without Rab27a silencing on STING/TBK1/IRF3 phosphorylation and fibrogenic marker expression in LX-2 cells. (j) Effect of the STING inhibitor SN-011 on LX-2 cells stimulated with conditioned media (CM) from cholesterol-treated hepatocytes. Data are expressed as the mean ± SEM of three independent experiments. Scale bar, 20 μm. Samples denoted by different letters (a, b and c) vary significantly ($p < 0.05$, one-way ANOVA followed by Tukey's post hoc test). $^{**}p < 0.01$ and $^{***}p < 0.001$.

Supplementary Fig. 5

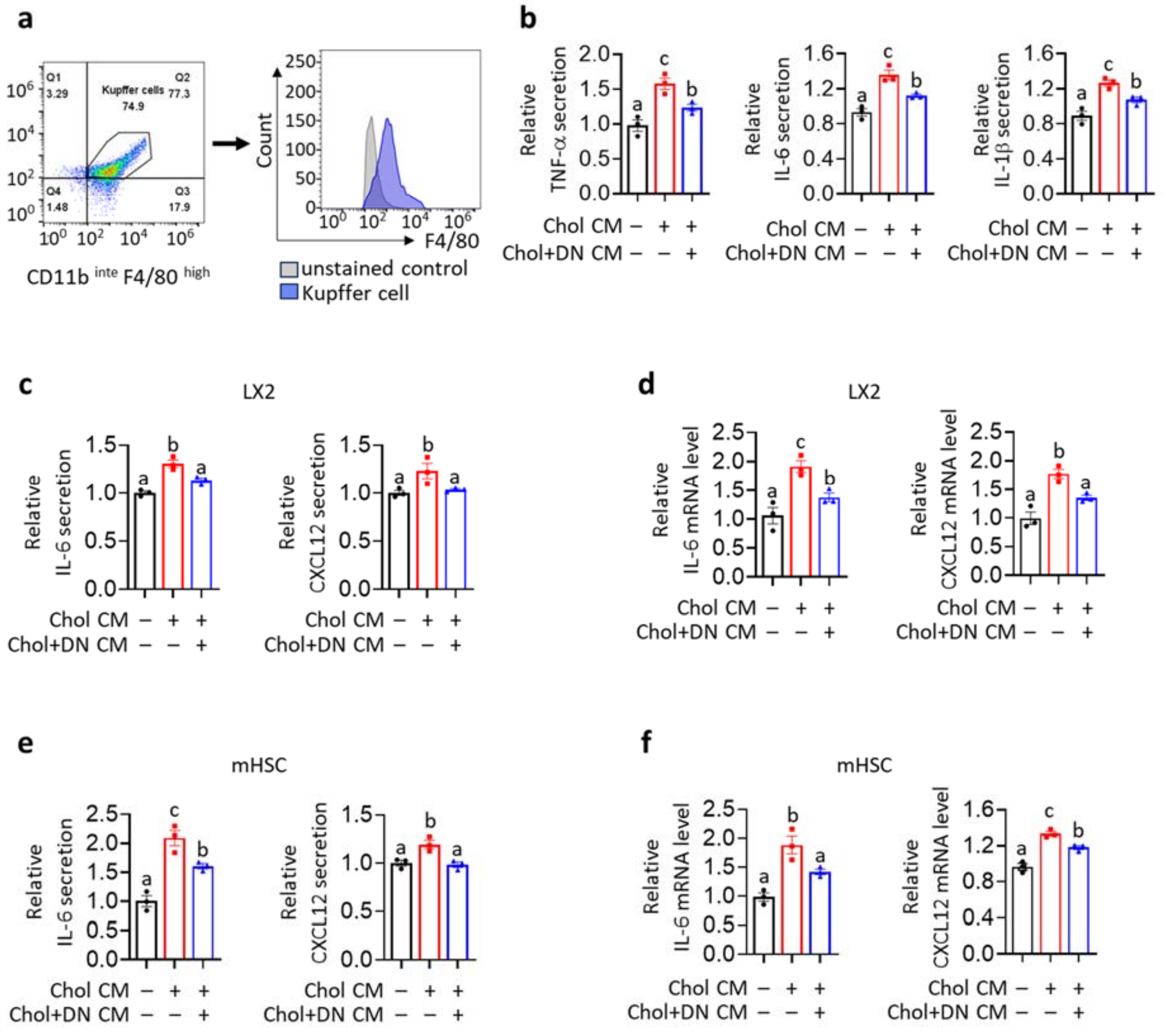

Supplementary Fig. 5. Effects of conditioned medium from DN203316-treated hepatocytes on inflammatory cytokine expression in Kupffer cells and HSCs. (a) Identification of Kupffer cells by flow-cytometric gating (CD11b^{int}, F4/80^{high}) (left panel), and gated cells with unstained control in an F4/80 histogram (right panel). (b) Effects of conditioned media (CM) from cholesterol-treated hepatocytes with or without DN203316 on inflammatory cytokine levels in Kupffer cells. (c–f) IL-6 and CXCL12 levels in hepatic stellate cells treated with CM from DN203316-mediated ferroptosis-inhibited hepatocytes. Data are expressed as the mean ± SEM of three independent experiments. Samples denoted by different

letters (a, b and c) vary significantly ($p < 0.05$, one-way ANOVA followed by Tukey's post hoc test).

Supplementary Fig. 6

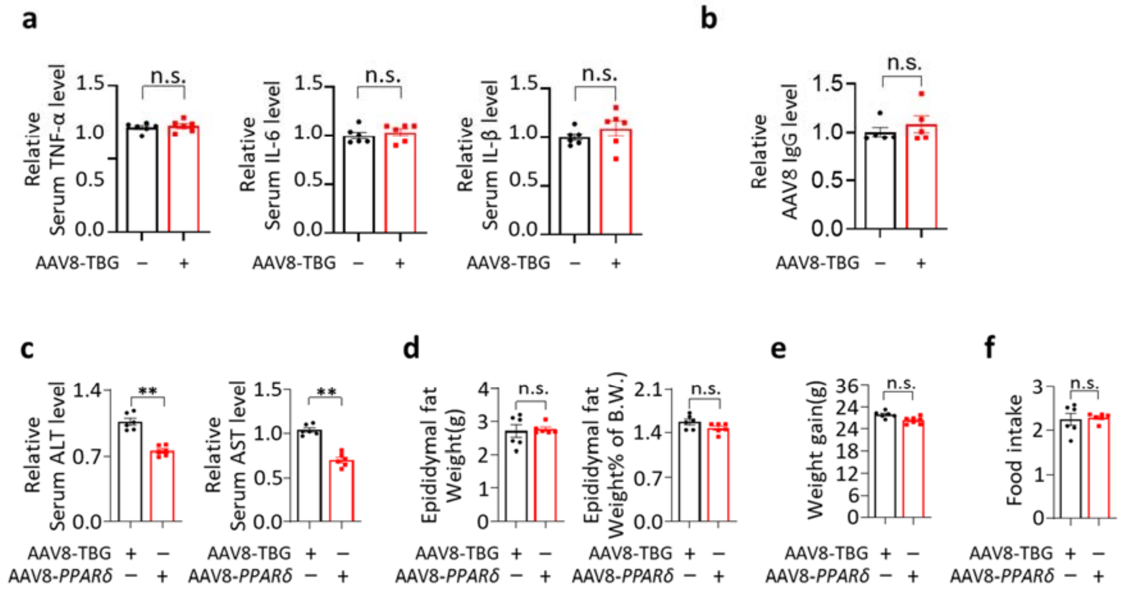

Supplementary Fig. 6. Effects of hepatocyte-specific PPARδ overexpression on metabolic parameters in an HFHC-induced MASH mouse model. (a, b) Serum levels of inflammatory cytokines (a) and AAV8-specific IgG (b) with or without AAV8-GFP vector injection. (c–f) Serum levels of ALT and AST (c), epididymal fat weight (d), body weight gain (e), and food intake (f) in HFHC-fed mice with or without hepatocyte-specific overexpression of PPARδ. Data are expressed as the mean ± SEM of three independent experiments. n.s.; not significant. ***p*< 0.01.

Supplementary Fig. 7

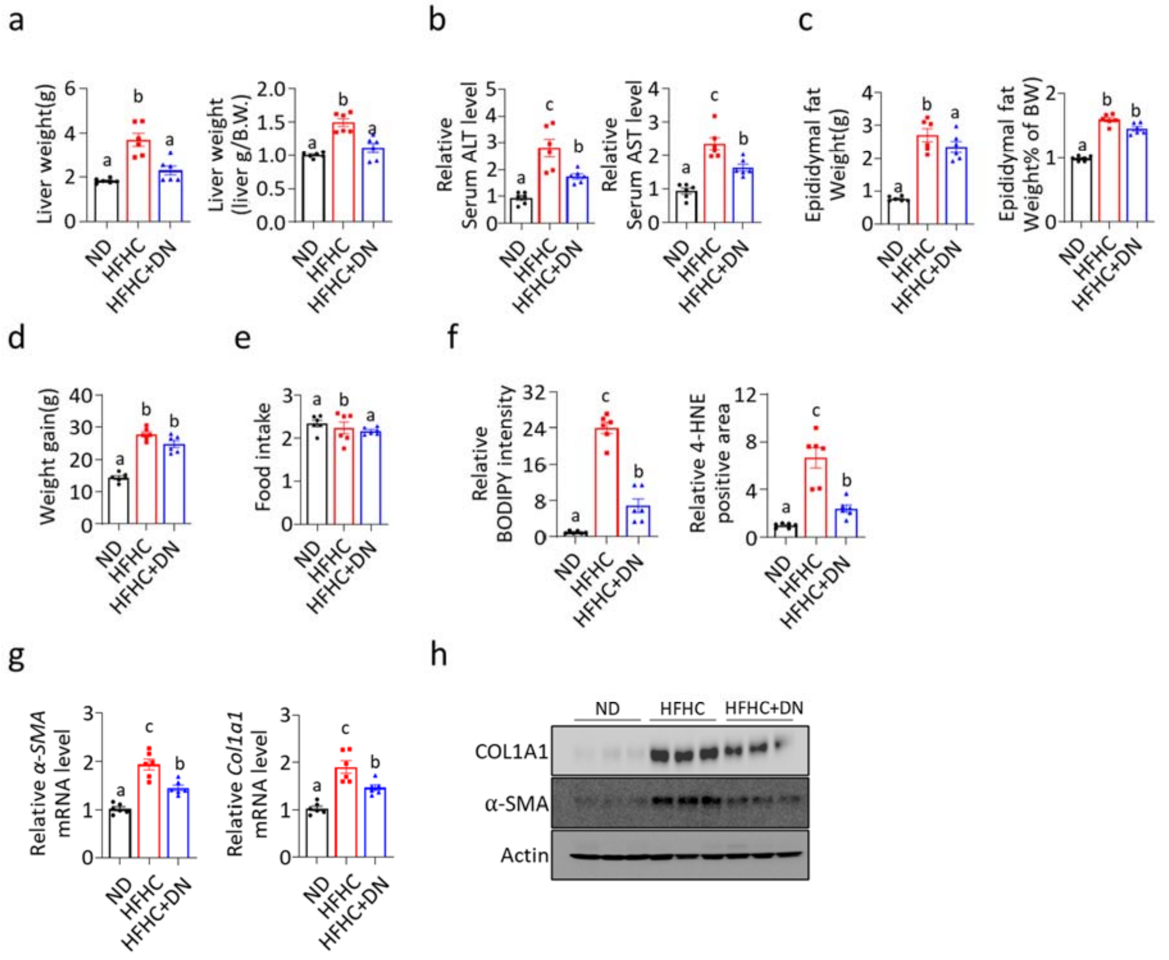

Supplementary Fig. 7. Effects of DN203316 on liver function and fibrosis in an HFHC-induced MASH mouse model. (a–e) Liver weight (a), serum ALT and AST levels (b), epididymal fat mass (c), body weight gain (d), and food intake (e) in HFHC-fed mice treated with or without DN203316 (n = 6 per group). (f) Quantitative analysis of the BODIPY and 4-HNE staining as shown in Fig. 6c (n = 6 per group). (g, h) mRNA expression (g) and protein levels (h) of fibrogenic markers in liver tissues from HFHC-fed mice treated with or without DN203316 (n = 6 per group). Samples denoted by different letters (a, b and c) vary significantly ($p < 0.05$, one-way ANOVA followed by Tukey's post hoc test).

Supplementary Table 1: Primer sequences for real-time PCR analysis

**Name**	**Sequence (5'- 3')**
m-*PPARδ*-F	GTCTCCGCAAGCCCTTCAG
m-*PPARδ*-R	TCCAGCGCATTGAACTTGAC
m-*xCT*-F	ATCTCCCCCAAGGGCATACT
m-*xCT*-R	CACAGGCAGACCAGAAAACCA
m-*GPX4*-F	CCTCCCCAGTACTGCAACAG
m-*GPX4*-R	GGCTGAGAATTCGTGCATGG
m-*α-SMA*-F	GTCCCAGACATCAGGGAGTAA
m-*α-SMA*-R	TCGGATACTTCAGCGTCAGGA
m-*Col1a1*-F	GGGGCAAGACAGTCATCGAA
m-*Col1a1*-R	GGG TGGAGGGAGTTTACACG
m-*IL-6*-F	TTCTCTGGGAAATCGTGGAAA
m-*IL-6*-R	TGCAAGTGCATCATCGTTGTT
m-*CXCL12*-F	AGAGCCAACGTCAAGCATCT
m-*CXCL12*-R	ATCTGAAGGGCACAGTTTGG
m-*GAPDH*-F	ACTCCACTCACGGCAAATTC
m-*GAPDH*-R	TCTCCATGGTGGTGAAGACA
h-*PPARδ*-F	GCAAGAAATGGGAAACATCCA
h-*PPARδ*-R	CCGTAAAGCCAAAGCTTCCA
h-*xCT*-F	TGGCCATTGTCACCATTGG
h-*xCT*-R	AGCAGCAGCTCCTCAGCATT
h-*GPX4*-F	GCTCCATGCACGAGTTTTCC
h-*GPX4*-R	ACACGAAGCCCCGGTACTT
h-*Rab27A*-F	TGGAGGACCAGAGAGTAGTGAAA
h-*Rab27A*-R	AGTTTCAAAGTAGGGGATTCCA
h-*α-SMA*-F	TTCAATGTCCCAGCCATGTA
h-*α-SMA*-R	GAAGGAATAGCCACGCTCAG
h-*Col1a1*-F	GAGGGCCAAGACGAAGACATC
h-*Col1a1*-R	CAGATCACGTCATCGCACAAC
h-*IL-6*-F	AGCTGCAGGCACAGAACCA
h-*IL-6*-R	AGCTGCGCAGAATGAGATGA

h-*CXCL12*-F	AGAGCCAACGTCAAGCATCT
h-*CXCL12*-R	ATCTGAAGGGCACAGTTTGG
h-*GAPDH*-F	CTGGGCTACACTGAGCACC
h-*GAPDH*-R	AAGTGGTCGTTGAGGGCAATG
h-mtDNA(ND)-F	ATA CCC ATG GCC AAC CTC CT
h-mtDNA(ND)-R	GGG CCT TTG CGT AGT TGT AT
